# Detection of driver mutations and genomic signatures in endometrial cancers using artificial intelligence algorithms

**DOI:** 10.1371/journal.pone.0299114

**Published:** 2024-02-26

**Authors:** Anda Stan, Korey Bosart, Mehak Kaur, Martin Vo, Wilber Escorcia, Ryan J. Yoder, Renee A. Bouley, Ruben C. Petreaca

**Affiliations:** 1 Biology Program, The Ohio State University, Marion, Ohio, United States of America; 2 Biology Department, Xavier University, Cincinnati, Ohio, United States of America; 3 Department of Chemistry and Biochemistry, The Ohio State University, Marion, Ohio, United States of America; 4 Department of Molecular Genetics, The Ohio State University, Marion, Ohio, United States of America; 5 James Comprehensive Cancer Center, The Ohio State University Columbus, Columbus, Ohio, United States of America; Avera Research Institute, UNITED STATES

## Abstract

Analyzed endometrial cancer (EC) genomes have allowed for the identification of molecular signatures, which enable the classification, and sometimes prognostication, of these cancers. Artificial intelligence algorithms have facilitated the partitioning of mutations into driver and passenger based on a variety of parameters, including gene function and frequency of mutation. Here, we undertook an evaluation of EC cancer genomes deposited on the Catalogue of Somatic Mutations in Cancers (COSMIC), with the goal to classify all mutations as either driver or passenger. Our analysis showed that approximately 2.5% of all mutations are driver and cause cellular transformation and immortalization. We also characterized nucleotide level mutation signatures, gross chromosomal re-arrangements, and gene expression profiles. We observed that endometrial cancers show distinct nucleotide substitution and chromosomal re-arrangement signatures compared to other cancers. We also identified high expression levels of the CLDN18 claudin gene, which is involved in growth, survival, metastasis and proliferation. We then used *in silico* protein structure analysis to examine the effect of certain previously uncharacterized driver mutations on protein structure. We found that certain mutations in CTNNB1 and TP53 increase protein stability, which may contribute to cellular transformation. While our analysis retrieved previously classified mutations and genomic alterations, which is to be expected, this study also identified new signatures. Additionally, we show that artificial intelligence algorithms can be effectively leveraged to accurately predict key drivers of cancer. This analysis will expand our understanding of ECs and improve the molecular toolbox for classification, diagnosis, or potential treatment of these cancers.

## Introduction

Cancers of the female reproductive organs can be generally classified into ovarian, uterine, cervical, vulvar, fallopian, and vaginal [1]. These cancers can occur in women of all ages but are more prevalent in older and post-menopausal women [2, 3]. Uterine cancers can be subdivided into endometrial cancers arising from the lining of the uterus and uterine sarcoma from uterine muscles [4]. Endometrial cancers (ECs) are the most common uterine cancers, are more aggressive than sarcoma, and have higher mortality [5].

ECs have historically been classified by estrogen status: Type I cancers are estrogen driven and occur in younger women, while Type II cancers are not driven by estrogen and occur in older women [6]. Type II cancers also tend to be more aggressive. Recent advances in genome sequencing and genetic characterization of cancer genomes driven primarily by The Cancer Genome Atlas (TCGA) has enabled classification of ECs by molecular status [7–9]. Four different EC molecular types are recognized: 1) POLE-mutant (ultra-mutated) showing mutations in the proofreading region of polymerase epsilon, one of the major replicative polymerases, 2) microsatellite instability (MSI+) characterized by mutations in mismatch repair genes, 3) microsatellite stable, and 4) copy number high/serous-like [10–13]. The latter two types are characterized by low mutation rates. Regardless of classification, all four types are distinguished by mutations of PTEN, PIK3CA, ARID1A, TP53, and KRAS genes, as well as other signal transduction, chromatin remodeling factors and histones being highly represented [11, 14–17].

The Catalogue of Somatic Mutations in Cancers (COSMIC) deposits analyzed cancer genomes data from both TCGA as well as other independent studies into a database [18]. Building on previous EC molecular data, we used rigorous artificial intelligence algorithms to classify all occurring mutations as either driver or passenger. *In silico* protein structure/function analyses were then employed to investigate how high frequency driver mutations affect protein structure and function. We also analyzed nucleotide substitution signatures, chromosomal re-arrangements, gene expression patterns, as well as other parameters with the goal to extract a more comprehensive genetic and genomic map for ECs.

## Materials and methods

### Genetic analysis

Complete mutation and chromosomal structural variation files were downloaded from COSMIC in Excel format. Gene expression data normalized as Z-values were also downloaded for TCGA samples.

S1B Table shows a listing between the percentages of total base pair mutations of the specified type among all types and the integer number of base pair mutations of the specified type. To compare nucleotide changes in endometrial vs. all cancers, additional data were extracted from COSMIC for all cancer tissues. Approximately 1200 genes were analyzed but only the nucleotide changes in the top 100 most mutated genes in endometrial cancers are shown in S1A Table. There are twelve different nucleotide substitution mutation possibilities: A>C, A>G, A>T, C>A, C>T, C>G, G>A, G>C, G>T, T>A, T>C, T>G. The percentages for every case of the twelve cases of base pair mutations were recorded. Twelve columns were created for the added-up integer values in the categories for all twelve nucleotide substitution mutation possibilities. Next an Independent Samples T-test was performed to compute significant probabilities (shown in red in S1C Table).

Descriptions for the functions of the most mutated genes in endometrial cancers were extracted from NCBI (S3 Table).

The STRING database (https://string-db.org/) was used to identify connections between genes and proteins. The database mines other databases and extracts validated connections (both physical and genetic) and makes computational predictions about the strength of connections and provides a score. The higher the score, the higher the likelihood that a meaningful connection exists. S1F Table only shows those pathways with a strength score of 0.1 or higher.

Driver and passenger mutations were classified using the OpenCRAVAT CHASM tool [19, 20]. To create valid input files from the COSMIC database to be used in OpenCRAVAT, Python was used to write a code that took an input.csv file containing genomic mutation data and processed it into a format suitable for analysis by the OpenCRAVAT tool. First, the.csv was converted into a tab separated.tsv file (csv_2_tsv), which provided an intermediate file for parsing. Next, the TSV file was filtered to extract only the HGVSG column containing the variant annotations (tsv_2_HGVSG). The HGVSG strings were parsed via regular expressions to pull out key details—chromosome, position, reference base, alternate base—into variables (HGVSG_2_CRAVAT). These components were written line-by-line into a new.tsv file in the specific columns required by OpenCRAVAT. By automating these sequential steps of file conversion, parsing, and reformatting, the original CSV data was transformed into the proper format for computational mutation analysis by OpenCRAVAT. The full pipeline was executed by calling csv_2_CRAVAT. This provided an efficient methodology for preparing large genomics datasets for downstream research applications. Link to the GitHub can be found here: https://github.com/dhakxls/cosmic-parser.

### Gene expression analysis

COSMIC reports gene expression levels for certain TCGA studies obtained either from microarray analysis or RNA-seq as a Z-value with values above Z = 2 considered over-expressed and under Z = -2 under-expressed while a value between -2 and 2 is interpreted as normal expression [21, 22]. We extracted gene expression data for all TCGA samples and computed the average Z-value expression change (S2 Table). S2 Fig shows those genes with a Z change greater than than 2. There were no changes with a score lower than -2.

### Effect of driver mutations on protein structure

The PDB files of the wild-type PPP2R1A (PDB ID: 1B3U) [23], PTEN (PDB ID: 1D5R) [24], PIK3CA (PDB ID: 2RD0) [25], CTNNB1 (PDB ID: 6M90) [26], and TP53 (PDB ID: 8F2I) [27] human protein 3D structures were downloaded from the Protein Data Bank. There was not a published crystal structure for human FGFR2 protein available on the Protein Databank website, so an AlphaFold [28, 29] model was downloaded instead. Amino acid mutations were made computationally using the mutagenesis function in the PyMOL Molecular Graphics System, Version 2.5.5 Schrödinger, LLC. Side chain polar interactions in a 4 Å radius were selected using PyMOL for the wild-type and mutant residue. All six protein PDB wild-type (WT) files were uploaded to the BRENDA Enzyme Database website and the stability of point mutations in comparison to WT were calculated using BRENDA’s CUPSAT calculation tool [30]. To analyze the electrostatic surface potential maps of the selected driver mutations, the PyMOL program with the APBS electrostatics plugin was used to visualize change between WT protein and driver mutated protein [31]. The localized area of the target mutation sequence was used as the center point for all observation and analysis.

All figures were made in Photoshop or PowerPoint.

## Results and discussion

### Mutation distribution in endometrial cancers

**Fig 1:**
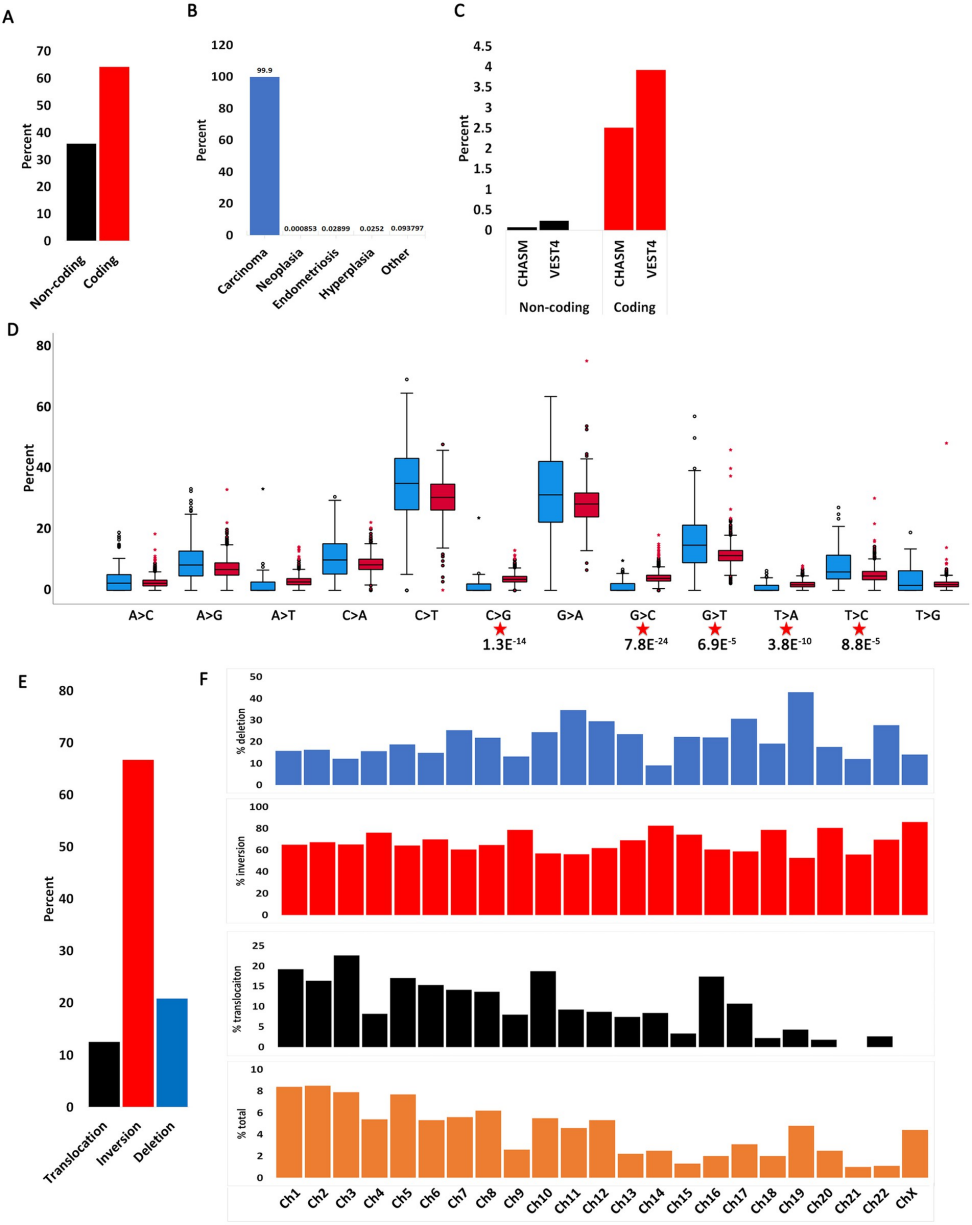
Mutation distribution in endometrial cancers. **A**. Percent coding and non-coding mutations in endometrial cancers. **B**. Distribution of coding mutations by endometrial cancer histology. **C**. Percent coding driver (CHASM) and pathogenic (VEST4) mutations out of total mutations in **A** in endometrial cancers. **D**. Distribution of nucleotide substitutions in coding endometrial mutations (blue) compared with coding mutations in other cancers (red). Significant p values between the two samples are shown (t-test: independent samples, unequal variance, two tails). **E**. Chromosomal re-arrangements in endometrial cancers. Data extracted using the CONNAN function on COSMIC.

Genetic mutation can occur in coding regions (translated into proteins) and non-coding regions (untranslated). COSMIC reports both coding and non-coding mutations with the caveat that non-coding represents only 5’ and 3’ UTRs and intronic rather than all “junk” DNA. About 65% of observed mutations are reported in coding regions with the remaining approximately 35% in non-coding regions (Fig 1A). Both coding and non-coding mutations have the potential to drive cellular transformation and immortalization. Coding mutations can directly affect protein/enzyme function while non-coding mutations can affect gene expression or splicing. When we characterized all forms of EC histology, we observed that most coding mutations occur in carcinoma (Fig 1B, S1 Fig).

Not all mutations affect gene function equally. For example, non-silent mutations introduce a direct change in amino acid sequence (e.g., missense, non-sense, InDel) and are predicted to have a higher impact on gene function than silent mutations. However, certain non-silent mutations have a more profound effect on gene function than others. For example, a missense mutation in the active site of an enzyme would be predicted to have a greater effect on gene function than a mutation elsewhere in its sequence. To understand which mutations are most likely to drive ECs, we used two ‘gold standard’ artificial intelligence algorithms: *C*ancer-Specific *H*igh-Throughput *A*nnotation of *S*omatic *M*utations (CHASMplus) [20] and the *V*ariant *E*ffect *S*coring *T*ool (VEST4) [32]. CHASM can classify missense mutations as driver or passenger while VEST4 predicts the probability that they are pathogenic. The algorithm computes a score and p-value. A p-value below 0.05 is considered statistically significant (e.g. mutation is driver). Using this approach, we distilled all EC mutations to only those predicted to have a major role in transformation and immortalization. This analysis shows that only about 2.5% of all coding mutations are predicted to be driver and about 4% pathogenic, while fewer than 0.5% of all non-coding mutations are likely to be either driver or pathogenic (Fig 1C, S1A Table). This indicates that most mutations in ECs are probably passenger mutations and may play minor roles in cellular transformation or immortalization.

We next examined the type of point mutations arising in ECs (Fig 1D, S1B Table). A previous report has investigated mutation signatures in endometrial adenocarcinoma and found that A:T>T:A and G:C>C:G are increased in proficient MMR cancers compared with deficient MMR [33]. Here, we analyzed each transversion and transition independently and compared the frequency of each nucleotide change in ECs to the frequency in other cancers (independent samples t-test). The goal was to understand whether EC mutations show a pattern different from other cancers. All comparisons produced significant p-values suggesting that ECs have unique mutation signatures than other cancers (S1C Table). As expected, G>A and C>T mutations occur at a higher frequency than the other mutations [33]. However, five mutation types (C>G, G>C, G>T, T>A, T>C) show a statistically significant higher frequency compared to other cancers. Of these, the C>G and G>C transversions are statistically more represented in ECs than other cancers (p = 1.3E^-14^ and p = 7.8E^-24^, respectively). The significance of this is not immediately obvious but the high levels of both changes suggests that they are replication rather than transcription driven (e.g., they occur on both DNA strands). Additionally, this cannot be due to MMR status because 1) previous findings showed that deficient MMR ECs have fewer G:C>C:G mutations [33] and 2) most cancers used for this comparison are not MMR unstable. Thus, the increase in the G:C>C:G signature is unique to ECs. Conversely, there is a higher level of G>T than C>A changes which indicates a strand bias. When these data are integrated with previous studies, there appears to be a unique mutational signature in ECs.

We next interrogated global chromosomal re-arrangements in ECs (Fig 1E and 1F, S1D Table). Chromosomal inversions account for almost 70% of all re-arrangements with deletions coming in second (about 20%) and translocations third (about 10%). When the data were partitioned by chromosome number, the longer chromosomes (e.g., 1–10) showed a higher level of inter-chromosomal re-arrangements (translocations) than the shorter chromosomes, which have a higher level of intra-chromosomal re-arrangements (e.g., deletions). The only five chromosomes not following this pattern are chromosomes 4, 9, 10, 16 and 19. Chromosomes 4 and 9 generally have low levels of re-arrangements and a decreased frequency of translocations. Conversely, chromosomes 10 and 16 have increased levels of translocations while chromosome 19 has a higher level of deletions. These data show that genome wide re-arrangements are not uniform on every chromosome, suggesting that re-arrangements on certain chromosomes are selected because they drive EC cellular transformation and immortalization.

### Gene expression changes in endometrial cancers

We also analyzed gene expression changes in endometrial samples. However, this analysis was somewhat restricted because gene expression was only available for TCGA samples and reported as a Z-score (please see Materials and methods). We identified a few genes with a tendency to be overexpressed (S2 Table, S2 Fig). Of note is the CLDN18 claudin gene which showed a generally high level of over-expression. High CLDN18 expression was recently reported in certain cervical adenocarcinomas [34]. Here, we show that it is generally over-expressed in all endometrial cancers, which agrees with previous conclusions [34] that it could serve as a molecular marker for ECs.

CLDN18 over-expression has been observed in several other tumors [35, 36], primarily gastric [37]. Two CLDN18 isoforms have been identified [38]. Claudiximab (also known as zolbetuximab) is an anti-CLDN18 antibody specific for isoform CLDN18.2 (isoform 2) [39]. The antibody is effective in promoting antibody- and complement-dependent cytotoxicity as reported in recent phase II clinical studies [39, 40]. CLDN18 isoform 2 CAR T-cell immunotherapy is another promising therapy for gastric cancers and also in clinical studies [41]. The finding that CLDN18 is also over-expressed in endometrial cancers highlight the importance of this gene and should be considered for similar targeted therapeutics.

### Genes and pathways affected by endometrial cancer driver mutations

**Fig 2:**
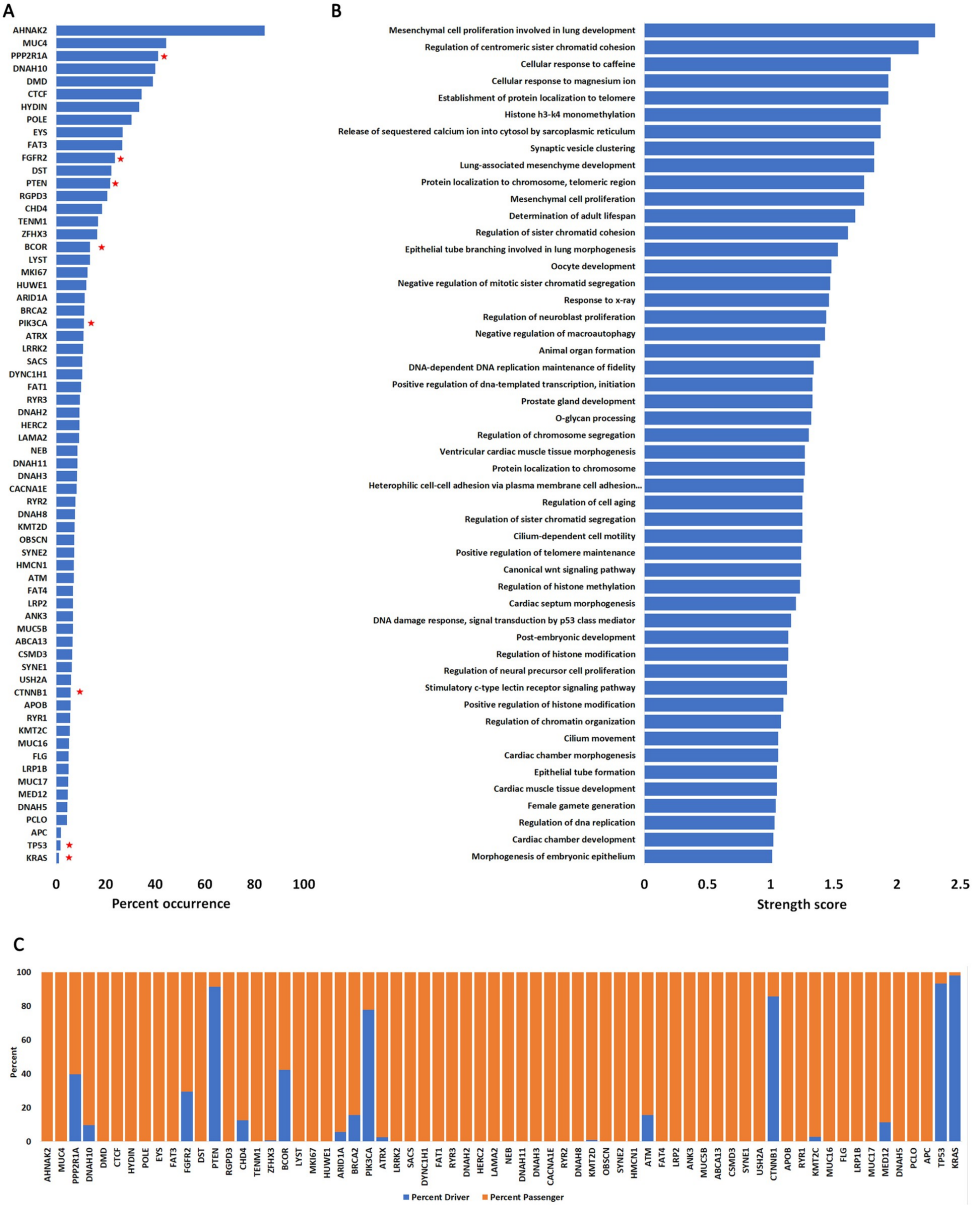
Genes and pathways affected by endometrial cancer mutations. **A.** Highly mutated genes detected in endometrial cancer patients. Shown are only those genes that are mutated in at least 100 patients out of 3213 (66 genes). For each of the 66 genes, we computed their occurrence in endometrial cancers compared with occurrence in all other cancers (expressed as percent). Red stars represent those genes with highest frequency of driver mutations. Complete data in S2 and S4 Tables. **B**. Biological processes affected by most frequent mutations in endometrial cancers. Only those processes with a string score above 0.1 are shown. **C**. Percent driver and passenger mutations for genes in **A** as determined by the CHASM algorithm.

Several previous publications have identified signature mutations in the endometrium (both normal and tumor) [11, 12, 17, 42–56]. Our goal here was to understand the significance of each mutation and to classify them as either driver or passenger (e.g., how likely are they to cause cellular transformation or immortalization). These driver mutations affect certain key pathways involved in cell proliferation, migration, and survival (Fig 5). We ran all mutations reported on COSMIC through two artificial intelligence algorithms: CHASM, which classifies mutations as either driver or passenger, and VEST4, which classifies mutations as pathogenic [20, 32]. We queried 3213 patients and identified driver mutations in 66 genes that occurred with higher frequency (at least 100 patients had the mutation) (Fig 2A, S1E Table). These mutations affect a variety of cellular pathways (Fig 2B, S1F Table) but DNA damage repair and checkpoint pathways were the most represented (S3 Fig, S3 Table).

Of the 66 genes identified, only 8 genes have a high level of driver mutations (30% or higher of total analyzed samples) (Fig 2A, S4 Table). Over 77% of residues mutated in five of these genes (PTEN, PIK3CA, CTNNB1, TP53 and KRAS) were characterized as driver and over 30% of mutated residues in three other genes (PPP2R1A, FGFR2, BCOR) were also characterized as driverSeveral others show a lower percentage of driver mutations (Fig 2C, S4 Table). Note that most of the EC mutated genes were not identified to harbor driver or pathogenic mutations indicating that they probably do not significantly contribute to cellular transformation.

**Fig 3:**
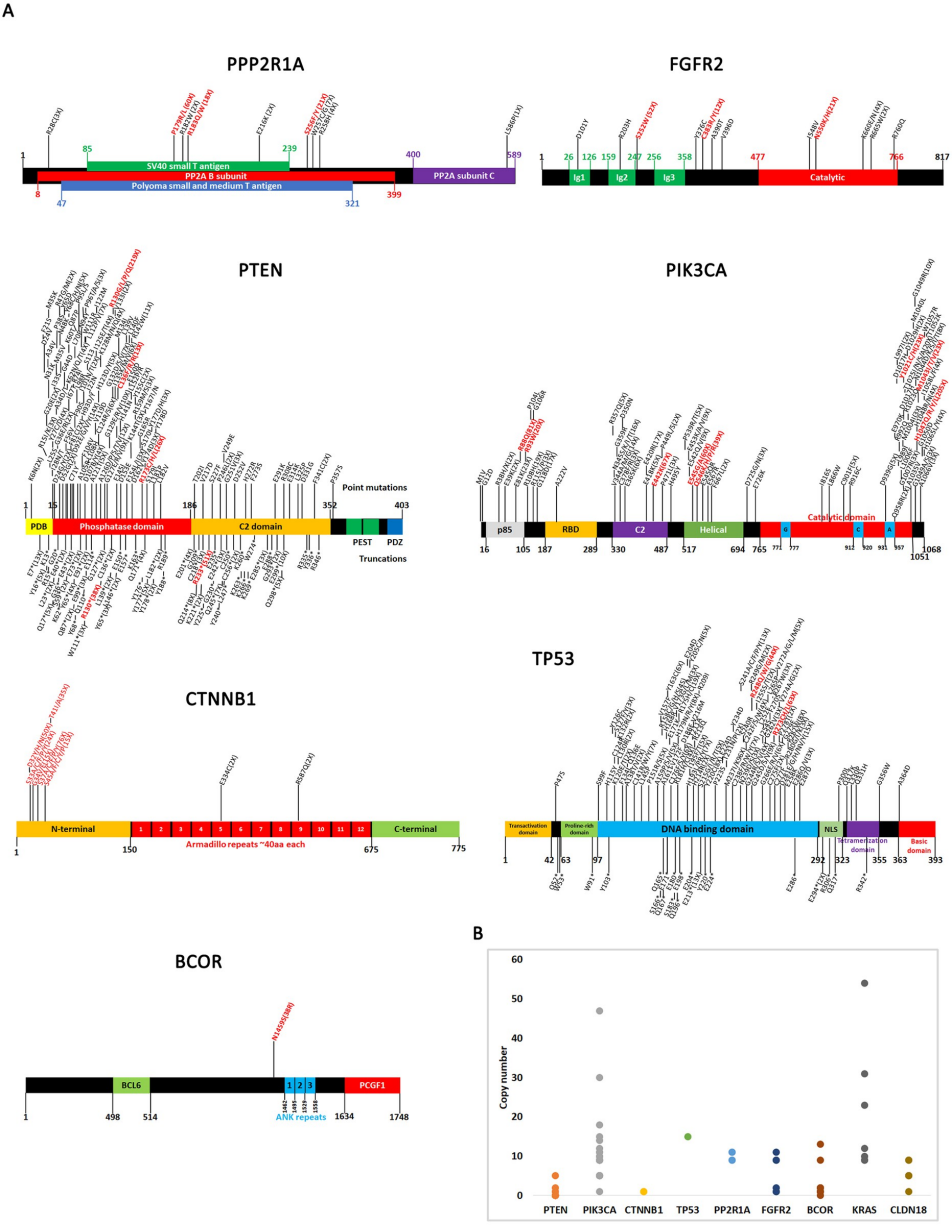
Driver mutations and copy number variations of most significantly altered genes. **A.** Domain diagrams of genes with location of driver mutations shown. Only driver mutations identified from CHASM are mapped onto these diagrams and the high frequency ones are indicated in red. The number of incidences of a certain mutation is shown in parentheses. All gene maps were made based on previous reports: PPP2R1A [58, 85–88], FGFR2 [73, 89], PTEN [90–94], PIK3CA [95, 96], CTNNB1 [68, 70, 97, 98], TP53 [99, 100], and BCOR [101]. **B**. Copy number variations of most significantly altered genes. Each dot represents one sample where high copy number was detected.

We next mapped all driver mutations on domain diagrams of the genes and indicated how many times each mutation was reported (Fig 3). A comprehensive literature search was also performed for each driver mutation to determine if these mutations have been previously identified and studied. In several cases, driver mutations identified from this study have been previously validated, which supports the ability of CHASM to correctly make predictions. A summary of previously published mutations is presented in S5 Table.

### Copy number variations and gene fusions

We also investigated copy number variations in the key driver genes (PTEN, PIK3CA, CTNNB1, TP53, PP2R1A, FGFR2, BCOR, KRAS, CLDN18)(Fig 3B). Not unexpectedly, we found that oncogenes (e.g. PIK3CA, KRAS) are characterized by high copy number in certain patients whereas tumor suppressor genes are generally low copy number. We also identified five patients with high copy numbers of the CLDN18 gene (three with 5 copies and two with 9 copies). This suggests that the high expression profiles for CLDN18 in certain patients may be due to allele duplication. COSMIC also reported one recurrent gene fusion, JAZF1-SUZ12, which has been previously identified and characterized [57].

### Effect of driver mutations on protein structure

Protein structural analysis was performed for all identified driver mutations that had not been previously studied in a similar way. Four mutations in PPP2R1A (P179R, R183W, S256F, and S256Y) had already been identified and analyzed in detail [58]. Two mutations in FGFR2, (N550K and N550H) were mapped onto a partial crystal structure [59]. Six mutations in PIK3CA were previously studied: R88Q [60], E545K and H1074R [61], and M1043I, M1043T, and M1043V [62]. Six mutations from PTEN (R130G, R130L, R130Q, R173H, and R173C) were computationally modeled and analyzed in detail [63]. Once the previously published driver mutations were removed, the 45 mutations that remained were modeled computationally using PyMOL with wild-type protein structures obtained from the Protein Data Bank [24–27, 64–66] or AlphaFold [65]. To study how these 45 mutations affect protein structure, a three-pronged approach was used in which side-chain tertiary interactions were analyzed, protein stability was predicted, and electrostatic surface potentials were calculated (S6 Table).

Polar contacts with the side chain of the WT and mutant residues were analyzed using PyMOL for all driver mutations. Mutations that reduced the number of polar contacts in comparison to WT were identified. There were 8 mutations out of 45 that reduced polar interactions (hydrogen bonds, dipole-dipole, and salt bridges) relative to WT (S4 Fig).

All mutations were also analyzed using CUPSAT to determine if they would affect protein stability, as determined by a ΔΔ**G** value [30]. The ΔΔ**G** is the difference in the Δ**G** of unfolding for the mutant protein and the WT protein, in which a negative value indicates destabilization of the protein structure, and a positive value indicates stabilization. The 10 mutations with the largest negative ΔΔ**G** values, representing the most destabilizing mutations, and the 10 with the largest positive ΔΔ**G** values, representing the most stabilizing mutations, were selected (Table 1). The protein that displayed the most mutations within this list of 20 was the CTNNB1 protein with 11 mutations (Table 1, S6 Table). CTNNB1 encodes the beta-catenin protein involved in the Wnt-signaling pathway with pleiotropic functions including cell proliferation and migration [67, 68]. Protein stability is regulated by GSK3-beta phosphorylation of several N-terminal residues (S33, S37, T41) which targets CTNNB1 for degradation (Fig 5) [69, 70]. CK1 also phosphorylates CTNNB1 at S45 and this posttranslational mark is also required for degradation. Not unexpectedly, mutations at these phosphorylated residues affect stability of the protein. Two other highly mutated residues in the N-terminus of the protein are D32 and G34 [71, 72]. Both residues are also required for CTNNB1 ubiquitination and mutations increase protein stability [72]. Remarkably, D32 and G34 decrease ubiquitination and degradation without affecting the phosphorylation status of S33, S37 and T41 [71]. Our analysis shows that the D32 and G34 mutations also affect general protein stability which may alter the function of the protein.

**Table 1:** Top 10 stabilizing or destabilizing mutations predicted to affect protein stability via CUPSAT organized by gene.

Gene/Protein	Mutation	Predicted ΔΔG (kcal/mol)	Predicted Effect
FGFR2	S252W	-12.66	Destabilizing
PTEN	C136R	-3.93	Destabilizing
PIK3CA	Y1021H	-1.66	Destabilizing
CTNNB1	D32N	4.38	Stabilizing
CTNNB1	D32H	-4.68	Destabilizing
CTNNB1	D32Y	-2.69	Destabilizing
CTNNB1	G34V	1.4	Stabilizing
CTNNB1	G34E	-8.24	Destabilizing
TP53	R248G	1.71	Stabilizing
TP53	R248Q	2.3	Stabilizing
TP53	R248W	4.65	Stabilizing
TP53	R273L	5.41	Stabilizing
TP53	R273C	17.86	Stabilizing
TP53	R273H	3.2	Stabilizing

FGFR2 is a receptor tyrosine kinase with pleiotropic functions including cell proliferation (Fig 5)[73]. The most destabilizing mutation was S252W, with a ΔΔG value of -12.66 kcal/mol. This is likely due to the significant change that takes place when mutating serine, which is a small polar residue, to tryptophan, a bulky aromatic hydrophobic residue. The S252 residue promotes ligand affinity and specificity [74]. The two most frequent mutations identified in endometrial cancers are FGFR2 S252W and N550K, which are both activating mutations [75, 76]. Our analysis shows that the S252W mutation also decreases protein stability.

The most stabilizing mutation was the TP53 R273C mutation with a ΔΔG of +17.86 kcal/mol. Mutation of R273 also displays oncogenic phenotypes [77] with various variants showing different oncogenic potential. Among all variants, the R273C mutation weakens DNA interactions and significantly affects TP53 function [78]. Our analysis shows that this change has a stabilizing effect on the TP53 protein, which may suggest that even in a heterozygous configuration, non-functional TP53 proteins with longer half-lives may outcompete functional ones. Interestingly, every TP53 driver mutation was identified as a stabilizing with a positive ΔΔG value. Every mutation of TP53 was also found to be in the top 20 mutations selected for their significance. Given that TP53 forms a tetrameric complex, stable non-functional alleles may outcompete functional alleles to form poorly functional complexes. No mutations within the PIK3CA protein nor the PPP2R1A were identified within either top 10 stabilizing or destabilizing mutations in Table 1.

**Fig 4:**
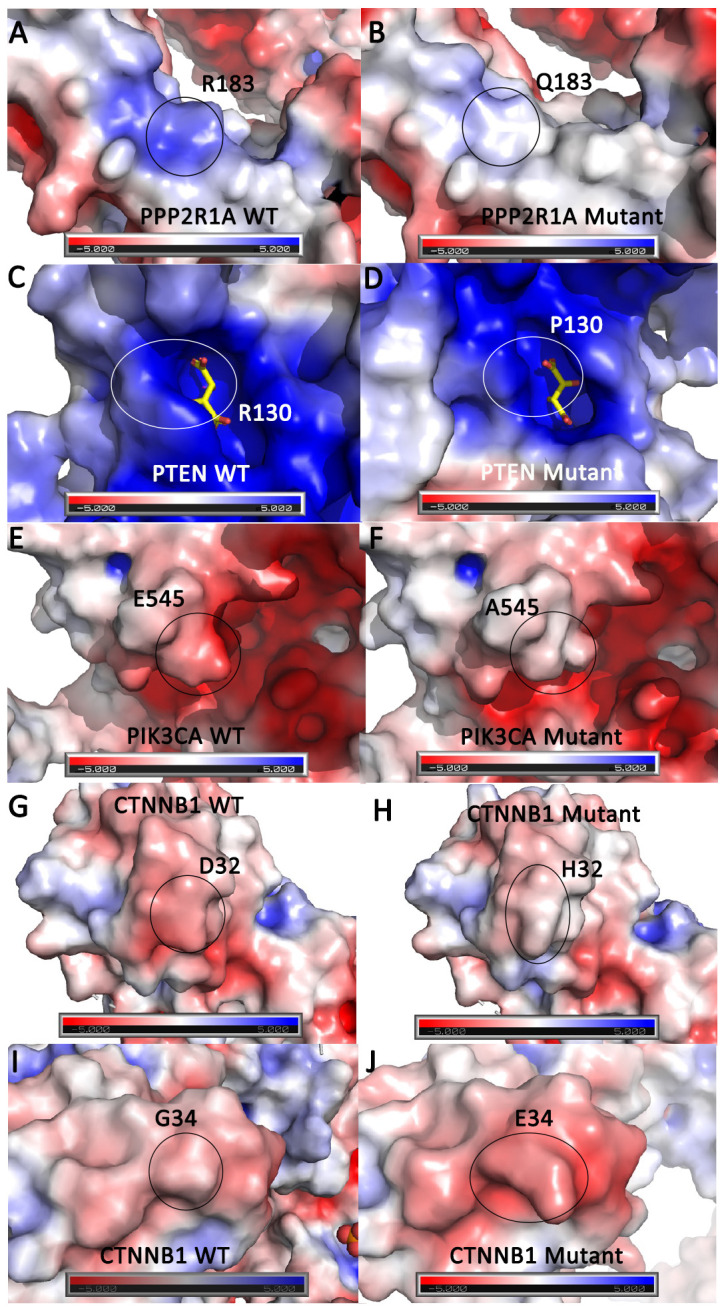
Top five driver mutations that alter protein electrostatic surface potential. Surface rendering of the protein structure is shown with basic or positive surface potential colored blue, acidic or negative colored red, and neutral colored white. The WT or mutant residue location is identified by a black or white circle. **A.** PPP2R1A WT R183 residue compared to **B.** PPP2R1A mutant Q183 residue. **C.** PTEN WT R130 residue with a tartrate molecule shown in yellow sticks compared to **D.** PTEN mutant P130 residue. **E.** PIK3CA WT E545 residue compared to **F.** PIK3CA mutant A545 residue. **G.** CTNNB1 WT D32 residue compared to **H.** CTNNB1 mutant H32 residue. **I.** CTNNB1 WT G34 residue compared to **J.** CTNNB1 mutant E34 residue.

Finally, electrostatic surface potential calculations were performed for the 12 mutations located on the exterior of the protein that were predicted to affect the pK_a_ or surface area based on the chemical properties of the amino acid side chains (S5 Fig). Mutations that were lectrostatically mapped were visually analyzed for observation of any color changes, which would indicate alterations in the local pK_a_, and for observation of any change in shape or size of the protein surface, which would indicate surface area alterations. The electrostatic change that occurred most often was the neutralization of a basic WT amino acid. This change is observationally recognized by a change from a blue electrostatic surface coloration into a white surface coloration. This neutralization of a basic environment occurred in 4 of the 12 total mutations. From the 12 mutations that were mapped, 5 mutations were selected that showed the most significant changes in surface coloration and/or by their alteration of WT surface area following mutation (Fig 4). The top 5 electrostatically altering mutations were scattered across PPP2R1A, PTEN, PIK3CA, and CTNNB1 proteins.

Using all the protein structure analysis data compiled, it was possible to identify which driver mutations can be classified as significantly affecting protein structure. This was performed by looking for overlapping mutations from the 8 mutations that reduced polar interactions, the top 10 stabilizing and top 10 destabilizing mutations with the largest change in ΔΔG value, and the 12 mutations that changed the electrostatic surface potential. Using this down-selection the total list of 45 studied driver mutations was narrowed down to 10 mutations (Table 2). All 10 of these mutations affected the electrostatic surface potential of the protein. Six of these 10 mutations reduced polar tertiary structure interactions as compared to WT. Seven of these 10 mutations were also identified in the either 20 mutations predicted to significantly affect protein stability (stabilizing or destabilizing) via CUPSAT.

**Table 2:** Mutations identified to significantly affect protein structure.

Protein	Mutation	Observed Electrostatic Change	Reduces Polar Interactions?	Predicted ΔΔG (kcal/mol)
FGFR2	S252W	Neutral to Neutral	No	-12.66
PTEN	R130P	Basic to Less Basic	Yes	-0.46
PIK3CA	E545A	Acidic to Neutral	Yes	-0.49
PIK3CA	E545G	Acidic to Neutral	Yes	-0.69
CTNNB1	D32H	Acidic to Neutral	No	-4.68
CTNNB1	G34E	Neutral to Acidic	No	-8.24
TP53	R273H	Basic to Neutral	Yes	3.2
TP53	R273C	Basic to Neutral	Yes	17.86
TP53	R273L	Basic to Less Basic	Yes	5.41

## Conclusion

Here, we analyzed genetic alterations in ECS. We employed gold standard artificial intelligence algorithms to characterize driver and passenger mutations. Our data agrees with previous findings, but we identified certain novel key mutation signatures in ECs that are distinct from other cancers. Of the 45 driver mutations analyzed using protein computational modeling, 10 mutations were identified as significantly effecting protein structure: FGFR2 (S252W), PTEN (R130P), PIK3CA (E545A, E545G), CTNNB1 (D32H, G34E, S37Y), and TP53 (R273H, R273C, R273L). Interestingly, the multiple mutations that were deemed significant in PIK3CA and TP53 were located at the same place in the primary sequence in each respective gene. The FGFR2 S252W mutation has been previously studied biochemically and shown to impact ligand binding and specificity [74]. The R130 residue in PTEN is located within the active site pocket and mutation of this residue has been implicated in various diseases and cancers [63, 79, 80]. Mutation of the PIK3CA E535 residue, specifically E535A, has been identified a predictive marker in breast cancer [81]. The S37 residue in CTNNB1 is a phosphorylation site and mutations have been found in colon cancers and melanoma [82, 83]. Finally, TP53 R273 variants generally show a loss of wild-type protein function [84].

**Fig 5:**
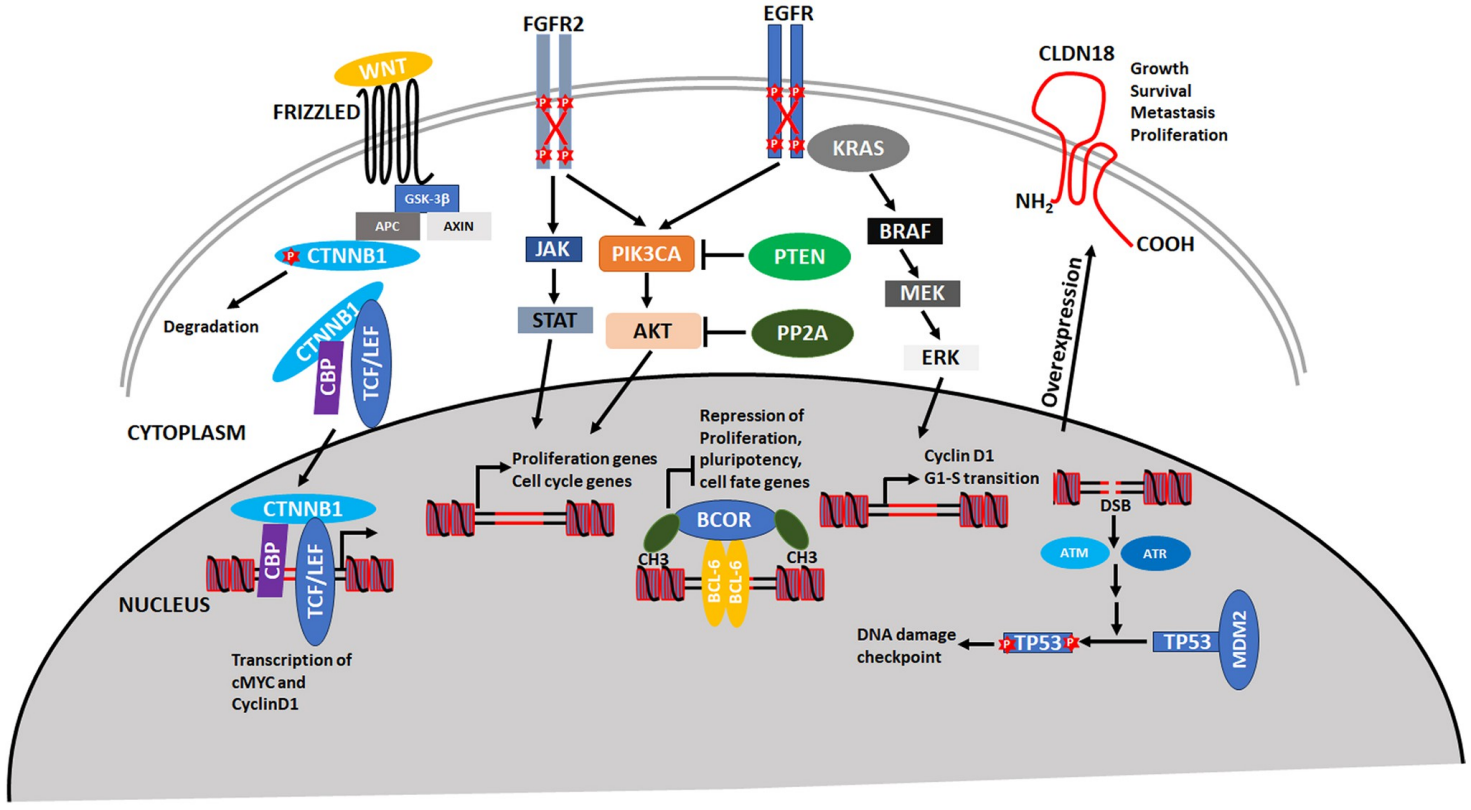
Major molecular pathways affected by driver mutations in endometrial cancers. Please see text for details and discussion.

The major molecular pathways affected by driver mutations in endometrial cancers are summarized in Fig 5. All pathways that were identified affect cellular proliferation and transformation. Mutations in CTNNB1 prevent its degradation and leads to formation of a stable transcriptional complex and activation of cMYC and Cyclin D1. The PIK3CA signal transduction pathway is one of the major pathways activated in endometrial cancers. PIK3CA can receive signals from both FGFR2 and EGFR, which results in activation of proliferation and cell cycle genes. In addition to activating mutations in FGFR2, EGFR and PIK3CA, loss of function mutations in the two phosphatases, PTEN and PP2A, leads to constitutive activation of this oncogenic pathway. Both FGFR2 and EGFR are key receptors that signal through PIK3CA as well as JAK-STAT and the MAPK pathways, respectively. Mutations in the BCOR transcriptional repressor activates pluripotency and cell fate genes. Loss of TP53 function has been well documented and is not unexpected because this gene is mutated in over 40% of cancers. An interesting finding is the over-expression of CLDN18 which has been demonstrated to activate metastasis and proliferation for other cancers but not endometrial cancers.

While many of the mutations investigated in this study have been previously published, our analysis sheds light on how these critical mutations impact protein structure. In addition, this study demonstrates that CHASM, which uses artificial intelligence, correctly predicts many driver mutations that have been experimentally validated. The data presented here could be used to refine ECs molecular signatures. These recurrent mutations and genomic alterations could be used as molecular markers for diagnosis and staging of ECs. Additionally, a comprehensive classification of driver mutations can aid in development of molecular therapies to target those genes and pathways that are key in EC cellular transformation and immortalization.

## Supporting information

S1 Fig Distribution of coding mutations in all EC histology types. Shown is frequency of all EC mutations by cancer histology. Endometrial carcinoma is the most represented histology in these data.
(PDF)

S2 Fig Over-expressed genes in endometrial cancers. Shown is a graph of average Z-values computed for all TCGA samples (from S2 Table).
(PDF)

S3 Fig Network interaction analysis of genes from Fig 2A. A map of network interactions using STRING database. Please see Materials and methods for this analysis.
(PDF)

S4 Fig Comparison of WT and mutant structures where a reduction in tertiary polar interactions was observed. Protein structures are shown with the mutated residue shown in orange. Interacting residues or small molecules are shown in yellow sticks. Polar interactions (salt bridge, hydrogen bond, or dipole-dipole) are shown as magenta dashed lines.
(PDF)

S5 Fig Mutations observed to change the electrostatic surface potential. Surface rendering of the protein structure is shown with basic or positive surface potential colored blue, acidic or negative colored red, and neutral colored white. The WT or mutant residue location is identified by a black or white circle.
(PDF)

S1 Table Gene and nucleotide level analysis in endometrial cancers. (XLSX)

S2 Table Z scores for all endometrial TCGA samples. (XLSX)

S3 Table Functions of most frequently mutated genes. Descriptions from NCBI.
(XLSX)

S4 Table Driver and passenger mutation analysis. (XLSX)

S5 Table Previously reported high frequency driver mutations. (DOCX)

S6 Table Polar and electrostatic analysis of selected mutations. (XLSX)

## References

[1] Centers for Disease Control and Prevention. (2023). Gynecological Cancers. https://www.cdc.gov/cancer/gynecologic/index.htm.

[2] Einstein M.H., Levine N.F., and Nevadunsky N.S., Menopause and Cancers. Endocrinol Metab Clin North Am, 2015. 44(3): p. 603–17. doi: 10.1016/j.ecl.2015.05.012 26316246

[3] Wu Y., et al., Age at Menopause and Risk of Developing Endometrial Cancer: A Meta-Analysis. Biomed Res Int, 2019. 2019: p. 8584130. doi: 10.1155/2019/8584130 31275987 PMC6560333

[4] Chelmow D., et al., Executive Summary of the Uterine Cancer Evidence Review Conference. Obstet Gynecol, 2022. 139(4): p. 626–643. doi: 10.1097/AOG.0000000000004711 35272316 PMC8936160

[5] Siegel R.L., Miller K.D., and Jemal A., Cancer statistics, 2020. CA Cancer J Clin, 2020. 70(1): p. 7–30. doi: 10.3322/caac.21590 31912902

[6] Guha P., et al., Estrogen receptors as potential therapeutic target in endometrial cancer. J Recept Signal Transduct Res, 2023. 43(1): p. 19–26. doi: 10.1080/10799893.2023.2187643 36883690

[7] Leon-Castillo A., Update in the molecular classification of endometrial carcinoma. Int J Gynecol Cancer, 2023. 33(3): p. 333–342. doi: 10.1136/ijgc-2022-003772 36878561

[8] Cancer Genome Atlas Research, N., et al., Integrated genomic characterization of endometrial carcinoma. Nature, 2013. 497(7447): p. 67–73. doi: 10.1038/nature12113 23636398 PMC3704730

[9] Talhouk A., et al., A clinically applicable molecular-based classification for endometrial cancers. Br J Cancer, 2015. 113(2): p. 299–310. doi: 10.1038/bjc.2015.190 26172027 PMC4506381

[10] Vermij L., et al., Incorporation of molecular characteristics into endometrial cancer management. Histopathology, 2020. 76(1): p. 52–63. doi: 10.1111/his.14015 31846532 PMC6972558

[11] Hong B., Le Gallo M., and Bell D.W., The mutational landscape of endometrial cancer. Curr Opin Genet Dev, 2015. 30: p. 25–31. doi: 10.1016/j.gde.2014.12.004 25622247 PMC4476916

[12] Temko D., et al., Somatic POLE exonuclease domain mutations are early events in sporadic endometrial and colorectal carcinogenesis, determining driver mutational landscape, clonal neoantigen burden and immune response. J Pathol, 2018. 245(3): p. 283–296. doi: 10.1002/path.5081 29604063 PMC6032922

[13] Kim T.M., Laird P.W., and Park P.J., The landscape of microsatellite instability in colorectal and endometrial cancer genomes. Cell, 2013. 155(4): p. 858–68. doi: 10.1016/j.cell.2013.10.015 24209623 PMC3871995

[14] O’Hara A.J. and Bell D.W., The genomics and genetics of endometrial cancer. Adv Genomics Genet, 2012. 2012(2): p. 33–47. doi: 10.2147/AGG.S28953 22888282 PMC3415201

[15] Kuhn E., et al., Identification of molecular pathway aberrations in uterine serous carcinoma by genome-wide analyses. J Natl Cancer Inst, 2012. 104(19): p. 1503–13. doi: 10.1093/jnci/djs345 22923510 PMC3692380

[16] Zhao S., et al., Mutational landscape of uterine and ovarian carcinosarcomas implicates histone genes in epithelial-mesenchymal transition. Proc Natl Acad Sci U S A, 2016. 113(43): p. 12238–12243. doi: 10.1073/pnas.1614120113 27791010 PMC5087050

[17] Momeni-Boroujeni A., et al., Landscape of chromatin remodeling gene alterations in endometrial carcinoma. Gynecol Oncol, 2023. 172: p. 54–64. doi: 10.1016/j.ygyno.2023.03.010 36958196 PMC10192087

[18] Tate J.G., et al., COSMIC: the Catalogue Of Somatic Mutations In Cancer. Nucleic Acids Res, 2019. 47(D1): p. D941–D947. doi: 10.1093/nar/gky1015 30371878 PMC6323903

[19] Pagel K.A., et al., Integrated Informatics Analysis of Cancer-Related Variants. JCO Clin Cancer Inform, 2020. 4: p. 310–317. doi: 10.1200/CCI.19.00132 32228266 PMC7113103

[20] Tokheim C. and Karchin R., CHASMplus Reveals the Scope of Somatic Missense Mutations Driving Human Cancers. Cell Syst, 2019. 9(1): p. 9–23 e8. doi: 10.1016/j.cels.2019.05.005 31202631 PMC6857794

[21] Cheadle C., et al., Analysis of microarray data using Z score transformation. J Mol Diagn, 2003. 5(2): p. 73–81. doi: 10.1016/S1525-1578(10)60455-2 12707371 PMC1907322

[22] Guo Y., et al., Large scale comparison of gene expression levels by microarrays and RNAseq using TCGA data. PLoS One, 2013. 8(8): p. e71462. doi: 10.1371/journal.pone.0071462 23977046 PMC3748065

[23] Groves M.R., et al., The structure of the protein phosphatase 2A PR65/A subunit reveals the conformation of its 15 tandemly repeated HEAT motifs. Cell, 1999. 96(1): p. 99–110. doi: 10.1016/s0092-8674(00)80963-0 9989501

[24] Lee J.O., et al., Crystal structure of the PTEN tumor suppressor: implications for its phosphoinositide phosphatase activity and membrane association. Cell, 1999. 99(3): p. 323–34. doi: 10.1016/s0092-8674(00)81663-3 10555148

[25] Huang C.H., et al., The structure of a human p110alpha/p85alpha complex elucidates the effects of oncogenic PI3Kalpha mutations. Science, 2007. 318(5857): p. 1744–8. doi: 10.1126/science.1150799 18079394

[26] Simonetta K.R., et al., Prospective discovery of small molecule enhancers of an E3 ligase-substrate interaction. Nat Commun, 2019. 10(1): p. 1402. doi: 10.1038/s41467-019-09358-9 30926793 PMC6441019

[27] Solares M.J., et al., High-Resolution Imaging of Human Cancer Proteins Using Microprocessor Materials. Chembiochem, 2022. 23(17): p. e202200310. doi: 10.1002/cbic.202200310 35789183 PMC9574649

[28] Kaboli Kafshgiri S., Farkhondeh T., and Miri-Moghaddam E., Glyphosate effects on the female reproductive systems: a systematic review. Rev Environ Health, 2022. 37(4): p. 487–500. doi: 10.1515/reveh-2021-0029 34265884

[29] Varadi M., et al., AlphaFold Protein Structure Database: massively expanding the structural coverage of protein-sequence space with high-accuracy models. Nucleic Acids Res, 2022. 50(D1): p. D439–D444. doi: 10.1093/nar/gkab1061 34791371 PMC8728224

[30] Parthiban V., Gromiha M.M., and Schomburg D., CUPSAT: prediction of protein stability upon point mutations. Nucleic Acids Res, 2006. 34(Web Server issue): p. W239–42. doi: 10.1093/nar/gkl190 16845001 PMC1538884

[31] Jurrus E., et al., Improvements to the APBS biomolecular solvation software suite. Protein Sci, 2018. 27(1): p. 112–128. doi: 10.1002/pro.3280 28836357 PMC5734301

[32] Douville C., et al., Assessing the Pathogenicity of Insertion and Deletion Variants with the Variant Effect Scoring Tool (VEST-Indel). Hum Mutat, 2016. 37(1): p. 28–35. doi: 10.1002/humu.22911 26442818 PMC5057310

[33] Lee P.J., et al., Clinical Targeted Next-Generation Sequencing Shows Increased Mutational Load in Endometrioid-type Endometrial Adenocarcinoma With Deficient DNA Mismatch Repair. Int J Gynecol Pathol, 2018. 37(6): p. 581–589. doi: 10.1097/PGP.0000000000000459 29084048

[34] Du X., et al., Membranous and nuclear staining of CLDN18 in HPV-independent and HPV-associated endocervical adenocarcinomas. Cancer Med, 2023. 12(2): p. 1441–1450. doi: 10.1002/cam4.5029 35861118 PMC9883430

[35] Cao W., et al., Claudin18.2 is a novel molecular biomarker for tumor-targeted immunotherapy. Biomark Res, 2022. 10(1): p. 38. doi: 10.1186/s40364-022-00385-1 35642043 PMC9153115

[36] Kyuno D., et al., Claudin-18.2 as a therapeutic target in cancers: cumulative findings from basic research and clinical trials. Tissue Barriers, 2022. 10(1): p. 1967080. doi: 10.1080/21688370.2021.1967080 34486479 PMC8794250

[37] Depotte L., et al., New developments and standard of care in the management of advanced gastric cancer. Clin Res Hepatol Gastroenterol, 2023. 48(1): p. 102245. doi: 10.1016/j.clinre.2023.102245 37952913

[38] Tureci O., et al., Claudin-18 gene structure, regulation, and expression is evolutionary conserved in mammals. Gene, 2011. 481(2): p. 83–92. doi: 10.1016/j.gene.2011.04.007 21571049

[39] Sahin U., et al., FAST: a randomised phase II study of zolbetuximab (IMAB362) plus EOX versus EOX alone for first-line treatment of advanced CLDN18.2-positive gastric and gastro-oesophageal adenocarcinoma. Ann Oncol, 2021. 32(5): p. 609–619. doi: 10.1016/j.annonc.2021.02.005 33610734

[40] Singh P., Toom S., and Huang Y., Anti-claudin 18.2 antibody as new targeted therapy for advanced gastric cancer. J Hematol Oncol, 2017. 10(1): p. 105. doi: 10.1186/s13045-017-0473-4 28494772 PMC5427576

[41] Grizzi G., et al., Anti-Claudin Treatments in Gastroesophageal Adenocarcinoma: Mainstream and Upcoming Strategies. J Clin Med, 2023. 12(8). doi: 10.3390/jcm12082973 37109309 PMC10142079

[42] Anglesio M.S., et al., Cancer-Associated Mutations in Endometriosis without Cancer. N Engl J Med, 2017. 376(19): p. 1835–1848. doi: 10.1056/NEJMoa1614814 28489996 PMC5555376

[43] Li X., et al., Whole-exome sequencing of endometriosis identifies frequent alterations in genes involved in cell adhesion and chromatin-remodeling complexes. Hum Mol Genet, 2014. 23(22): p. 6008–21. doi: 10.1093/hmg/ddu330 24969084

[44] Moore L., et al., The mutational landscape of normal human endometrial epithelium. Nature, 2020. 580(7805): p. 640–646. doi: 10.1038/s41586-020-2214-z 32350471

[45] Jamieson A. and McAlpine J.N., Molecular Profiling of Endometrial Cancer From TCGA to Clinical Practice. J Natl Compr Canc Netw, 2023. 21(2): p. 210–216. doi: 10.6004/jnccn.2022.7096 36791751

[46] Momeni-Boroujeni A., et al., Genomic landscape of endometrial carcinomas of no specific molecular profile. Mod Pathol, 2022. 35(9): p. 1269–1278. doi: 10.1038/s41379-022-01066-y 35365770 PMC9427676

[47] Hong J.H., et al., Genomic landscape of advanced endometrial cancer analyzed by targeted next-generation sequencing and the cancer genome atlas (TCGA) dataset. J Gynecol Oncol, 2022. 33(3): p. e29. doi: 10.3802/jgo.2022.33.e29 35128859 PMC9024183

[48] Choi J., et al., Distinct Genomic Landscapes in Early-Onset and Late-Onset Endometrial Cancer. JCO Precis Oncol, 2022. 6: p. e2100401. doi: 10.1200/PO.21.00401 35108035 PMC8820918

[49] Li L., et al., Genome-wide mutation analysis in precancerous lesions of endometrial carcinoma. J Pathol, 2021. 253(1): p. 119–128. doi: 10.1002/path.5566 33016334

[50] Kyo S., Sato S., and Nakayama K., Cancer-associated mutations in normal human endometrium: Surprise or expected? Cancer Sci, 2020. 111(10): p. 3458–3467. doi: 10.1111/cas.14571 32654393 PMC7541016

[51] Dou Y., et al., Proteogenomic Characterization of Endometrial Carcinoma. Cell, 2020. 180(4): p. 729–748 e26. doi: 10.1016/j.cell.2020.01.026 32059776 PMC7233456

[52] DeLair D.F., et al., The genetic landscape of endometrial clear cell carcinomas. J Pathol, 2017. 243(2): p. 230–241. doi: 10.1002/path.4947 28718916 PMC5708127

[53] Chang Y.S., et al., Identification of novel mutations in endometrial cancer patients by whole-exome sequencing. Int J Oncol, 2017. 50(5): p. 1778–1784. doi: 10.3892/ijo.2017.3919 28339086

[54] Jones N.L., et al., Distinct molecular landscapes between endometrioid and nonendometrioid uterine carcinomas. Int J Cancer, 2017. 140(6): p. 1396–1404. doi: 10.1002/ijc.30537 27905110

[55] Chang Y.S., et al., Genetic alterations in endometrial cancer by targeted next-generation sequencing. Exp Mol Pathol, 2016. 100(1): p. 8–12. doi: 10.1016/j.yexmp.2015.11.026 26626801

[56] Choi Y.J., et al., Genomic landscape of endometrial stromal sarcoma of uterus. Oncotarget, 2015. 6(32): p. 33319–28. doi: 10.18632/oncotarget.5384 26429873 PMC4741768

[57] Hrzenjak A., JAZF1/SUZ12 gene fusion in endometrial stromal sarcomas. Orphanet J Rare Dis, 2016. 11: p. 15. doi: 10.1186/s13023-016-0400-8 26879382 PMC4754953

[58] Taylor S.E., et al., The Highly Recurrent PP2A Aalpha-Subunit Mutation P179R Alters Protein Structure and Impairs PP2A Enzyme Function to Promote Endometrial Tumorigenesis. Cancer Res, 2019. 79(16): p. 4242–4257. 31142515 10.1158/0008-5472.CAN-19-0218 PMC6724736

[59] Dixit A. and Verkhivker G.M., Structure-functional prediction and analysis of cancer mutation effects in protein kinases. Comput Math Methods Med, 2014. 2014: p. 653487. doi: 10.1155/2014/653487 24817905 PMC4000980

[60] Jin N., et al., Therapeutic implications of activating noncanonical PIK3CA mutations in head and neck squamous cell carcinoma. J Clin Invest, 2021. 131(22). doi: 10.1172/JCI150335 34779417 PMC8592538

[61] Miller M.S., et al., Structural basis of nSH2 regulation and lipid binding in PI3Kalpha. Oncotarget, 2014. 5(14): p. 5198–208. 25105564 10.18632/oncotarget.2263 PMC4170646

[62] Gymnopoulos M., Elsliger M.A., and Vogt P.K., Rare cancer-specific mutations in PIK3CA show gain of function. Proc Natl Acad Sci U S A, 2007. 104(13): p. 5569–74. doi: 10.1073/pnas.0701005104 17376864 PMC1838453

[63] Smith I.N. and Briggs J.M., Structural mutation analysis of PTEN and its genotype-phenotype correlations in endometriosis and cancer. Proteins, 2016. 84(11): p. 1625–1643. doi: 10.1002/prot.25105 27481051 PMC5073044

[64] Ponomarenko S., Volfson I., and Strotmann H., Proton gradient-induced changes of the interaction between CF0 and CF1 related to activation of the chloroplast ATP synthase. FEBS Lett, 1999. 443(2): p. 136–8. doi: 10.1016/s0014-5793(98)01681-0 9989591

[65] Jumper J., et al., Highly accurate protein structure prediction with AlphaFold. Nature, 2021. 596(7873): p. 583–589. doi: 10.1038/s41586-021-03819-2 34265844 PMC8371605

[66] Wu W., et al., The influence of natural weathering on the behavior of heavy metals in small basaltic watersheds: A comparative study from different regions in China. Chemosphere, 2021. 262: p. 127897. doi: 10.1016/j.chemosphere.2020.127897 32791371

[67] Pai S.G., et al., Wnt/beta-catenin pathway: modulating anticancer immune response. J Hematol Oncol, 2017. 10(1): p. 101. doi: 10.1186/s13045-017-0471-6 28476164 PMC5420131

[68] Gottardi C.J. and Gumbiner B.M., Adhesion signaling: how beta-catenin interacts with its partners. Curr Biol, 2001. 11(19): p. R792–4. doi: 10.1016/s0960-9822(01)00473-0 11591340

[69] Li H., Pamukcu R., and Thompson W.J., beta-Catenin signaling: therapeutic strategies in oncology. Cancer Biol Ther, 2002. 1(6): p. 621–5. doi: 10.4161/cbt.309 12642683

[70] Ledinek Z., Sobocan M., and Knez J., The Role of CTNNB1 in Endometrial Cancer. Dis Markers, 2022. 2022: p. 1442441. doi: 10.1155/2022/1442441 35531470 PMC9072012

[71] Provost E., et al., Functional correlates of mutation of the Asp32 and Gly34 residues of beta-catenin. Oncogene, 2005. 24(16): p. 2667–76. doi: 10.1038/sj.onc.1208346 15829978

[72] Al-Fageeh M., et al., Phosphorylation and ubiquitination of oncogenic mutants of beta-catenin containing substitutions at Asp32. Oncogene, 2004. 23(28): p. 4839–46. doi: 10.1038/sj.onc.1207634 15064718 PMC2267883

[73] Gatius S., et al., FGFR2 alterations in endometrial carcinoma. Mod Pathol, 2011. 24(11): p. 1500–10. doi: 10.1038/modpathol.2011.110 21725289

[74] Willie D., et al., Cleft Palate in Apert Syndrome. J Dev Biol, 2022. 10(3). doi: 10.3390/jdb10030033 35997397 PMC9397066

[75] Pollock P.M., et al., Frequent activating FGFR2 mutations in endometrial carcinomas parallel germline mutations associated with craniosynostosis and skeletal dysplasia syndromes. Oncogene, 2007. 26(50): p. 7158–62. doi: 10.1038/sj.onc.1210529 17525745 PMC2871595

[76] Dutt A., et al., Drug-sensitive FGFR2 mutations in endometrial carcinoma. Proc Natl Acad Sci U S A, 2008. 105(25): p. 8713–7. doi: 10.1073/pnas.0803379105 18552176 PMC2438391

[77] Garg A., et al., Variable Mutations at the p53-R273 Oncogenic Hotspot Position Leads to Altered Properties. Biophys J, 2020. 118(3): p. 720–728. doi: 10.1016/j.bpj.2019.12.015 31952808 PMC7002923

[78] Li J., et al., Mutants TP53 p.R273H and p.R273C but not p.R273G enhance cancer cell malignancy. Hum Mutat, 2014. 35(5): p. 575–84. doi: 10.1002/humu.22528 24677579

[79] Chi A.S., et al., Prospective, high-throughput molecular profiling of human gliomas. J Neurooncol, 2012. 110(1): p. 89–98. doi: 10.1007/s11060-012-0938-9 22821383 PMC3583376

[80] Wang S.I., et al., Somatic mutations of PTEN in glioblastoma multiforme. Cancer Res, 1997. 57(19): p. 4183–6. 9331071

[81] Desriani and Al-Ahwani F., The sensitivity and efficacy method of PIK3CA exon 9 E545A as a high diagnostic accuracy in breast cancer. J Genet Eng Biotechnol, 2018. 16(1): p. 71–76. 30647707 10.1016/j.jgeb.2017.10.002 PMC6296600

[82] de La Coste A., et al., Somatic mutations of the beta-catenin gene are frequent in mouse and human hepatocellular carcinomas. Proc Natl Acad Sci U S A, 1998. 95(15): p. 8847–51. doi: 10.1073/pnas.95.15.8847 9671767 PMC21165

[83] Morin P.J., et al., Activation of beta-catenin-Tcf signaling in colon cancer by mutations in beta-catenin or APC. Science, 1997. 275(5307): p. 1787–90. doi: 10.1126/science.275.5307.1787 9065402

[84] Irshaid L., et al., Endometrial Carcinoma as the Presenting Malignancy in a Teenager With a Pathogenic TP53 Germline Mutation: A Case Report and Literature Review. Int J Gynecol Pathol, 2022. 41(3): p. 258–267. doi: 10.1097/PGP.0000000000000792 33990091

[85] Remmerie M. and Janssens V., PP2A: A Promising Biomarker and Therapeutic Target in Endometrial Cancer. Front Oncol, 2019. 9: p. 462. doi: 10.3389/fonc.2019.00462 31214504 PMC6558005

[86] Kauko O. and Westermarck J., Non-genomic mechanisms of protein phosphatase 2A (PP2A) regulation in cancer. Int J Biochem Cell Biol, 2018. 96: p. 157–164. doi: 10.1016/j.biocel.2018.01.005 29355757

[87] Jeong A.L., et al., Patient derived mutation W257G of PPP2R1A enhances cancer cell migration through SRC-JNK-c-Jun pathway. Sci Rep, 2016. 6: p. 27391. doi: 10.1038/srep27391 27272709 PMC4895347

[88] Shih Ie M., et al., Somatic mutations of PPP2R1A in ovarian and uterine carcinomas. Am J Pathol, 2011. 178(4): p. 1442–7. doi: 10.1016/j.ajpath.2011.01.009 21435433 PMC3078449

[89] Stehbens S.J., et al., FGFR2-activating mutations disrupt cell polarity to potentiate migration and invasion in endometrial cancer cell models. J Cell Sci, 2018. 131(15). doi: 10.1242/jcs.213678 30002137

[90] Molinari F. and Frattini M., Functions and Regulation of the PTEN Gene in Colorectal Cancer. Front Oncol, 2013. 3: p. 326. doi: 10.3389/fonc.2013.00326 24475377 PMC3893597

[91] Wang X. and Jiang X., PTEN: a default gate-keeping tumor suppressor with a versatile tail. Cell Res, 2008. 18(8): p. 807–16. doi: 10.1038/cr.2008.83 18626510

[92] Bell D.W. and Ellenson L.H., Molecular Genetics of Endometrial Carcinoma. Annu Rev Pathol, 2019. 14: p. 339–367. doi: 10.1146/annurev-pathol-020117-043609 30332563

[93] Markowska A., et al., Signalling pathways in endometrial cancer. Contemp Oncol (Pozn), 2014. 18(3): p. 143–8. doi: 10.5114/wo.2014.43154 25520571 PMC4268999

[94] Wu Y., et al., Significance of a PTEN Mutational Status-Associated Gene Signature in the Progression and Prognosis of Endometrial Carcinoma. Oxid Med Cell Longev, 2022. 2022: p. 5130648. doi: 10.1155/2022/5130648 35251475 PMC8890874

[95] Samuels Y., et al., High frequency of mutations of the PIK3CA gene in human cancers. Science, 2004. 304(5670): p. 554. doi: 10.1126/science.1096502 15016963

[96] Saal L.H., et al., PIK3CA mutations correlate with hormone receptors, node metastasis, and ERBB2, and are mutually exclusive with PTEN loss in human breast carcinoma. Cancer Res, 2005. 65(7): p. 2554–9. doi: 10.1158/0008-5472-CAN-04-3913 15805248

[97] Parrish M.L., Broaddus R.R., and Gladden A.B., Mechanisms of mutant beta-catenin in endometrial cancer progression. Front Oncol, 2022. 12: p. 1009345. 36248967 10.3389/fonc.2022.1009345 PMC9556987

[98] Travaglino A., et al., Prognostic significance of CTNNB1 mutation in early stage endometrial carcinoma: a systematic review and meta-analysis. Arch Gynecol Obstet, 2022. 306(2): p. 423–431. doi: 10.1007/s00404-021-06385-0 35034160 PMC9349085

[99] Tanaka T., Watanabe M., and Yamashita K., Potential therapeutic targets of TP53 gene in the context of its classically canonical functions and its latest non-canonical functions in human cancer. Oncotarget, 2018. 9(22): p. 16234–16247. doi: 10.18632/oncotarget.24611 29662640 PMC5882331

[100] Mirzayans R., et al., New insights into p53 signaling and cancer cell response to DNA damage: implications for cancer therapy. J Biomed Biotechnol, 2012. 2012: p. 170325. doi: 10.1155/2012/170325 22911014 PMC3403320

[101] Astolfi A., et al., BCOR involvement in cancer. Epigenomics, 2019. 11(7): p. 835–855. doi: 10.2217/epi-2018-0195 31150281 PMC6595546

