# Supplementary figures and images for: Detection of driver mutations and genomic signatures in endometrial cancers using artificial intelligence algorithms
Source: PLoS One. 2024 Feb 26;19(2):e0299114. doi: 10.1371/journal.pone.0299114 (PMC10896512; doi:10.1371/journal.pone.0299114)

Supplementary Figure S1. Distribution of coding mutations in all EC histology types.

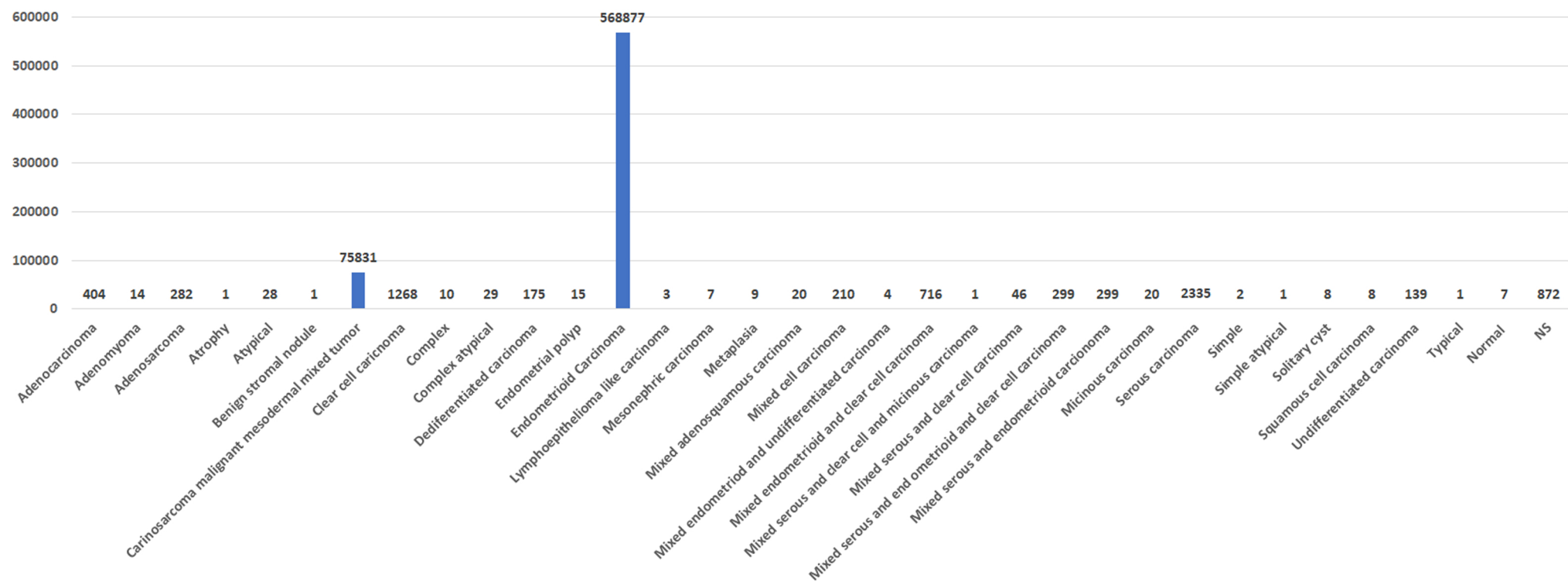

Supplement: S1 Fig — Shown is frequency of all EC mutations by cancer histology. Endometrial carcinoma is the most represented histology in these data. (PDF) [file pone.0299114.s001.pdf]

Supplementary Figure S2. Over-expressed genes in TCGA endometrial cancer samples.

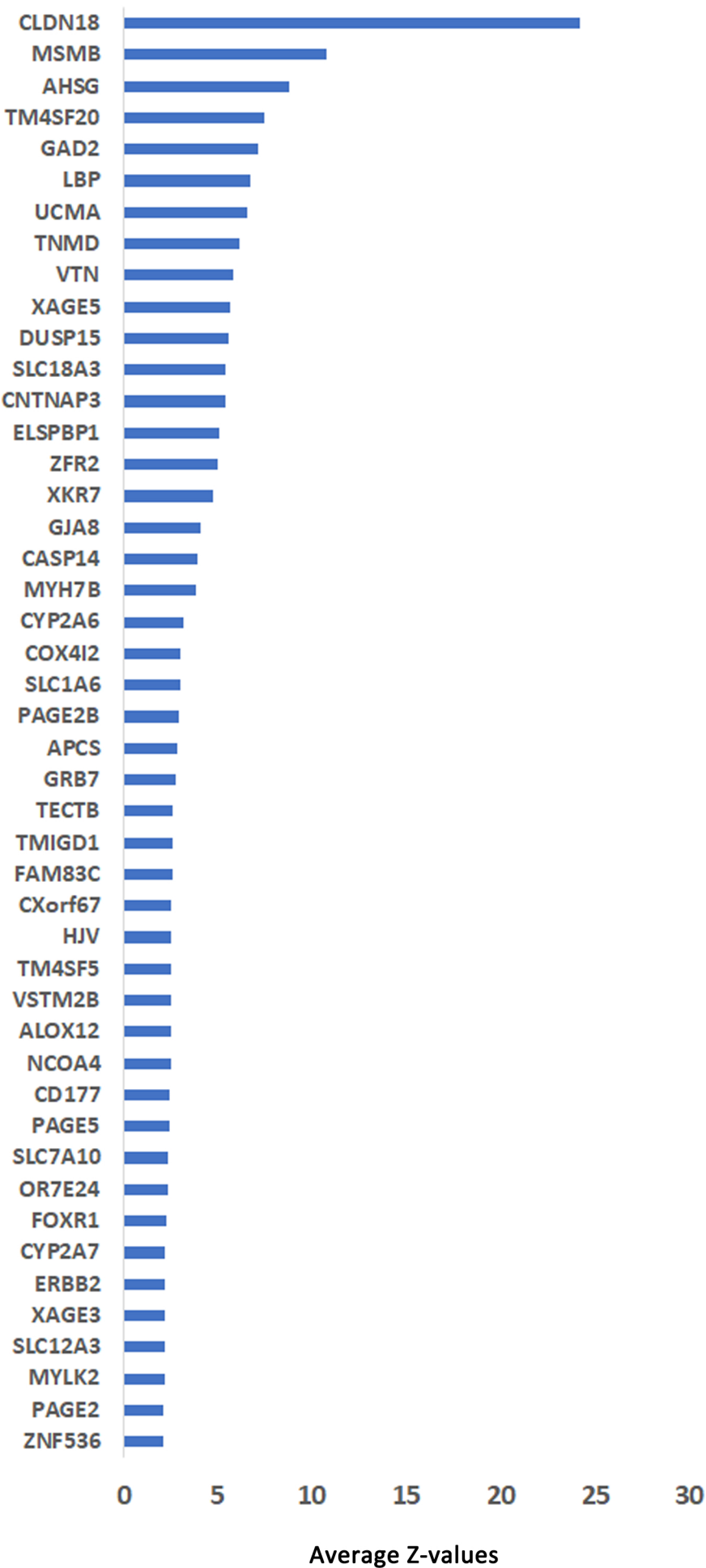

Supplement: S2 Fig — Shown is a graph of average Z-values computed for all TCGA samples (from S2 Table). (PDF) [file pone.0299114.s002.pdf]

Supplementary Figure S3. Network interaction analysis of genes from Figure 2A

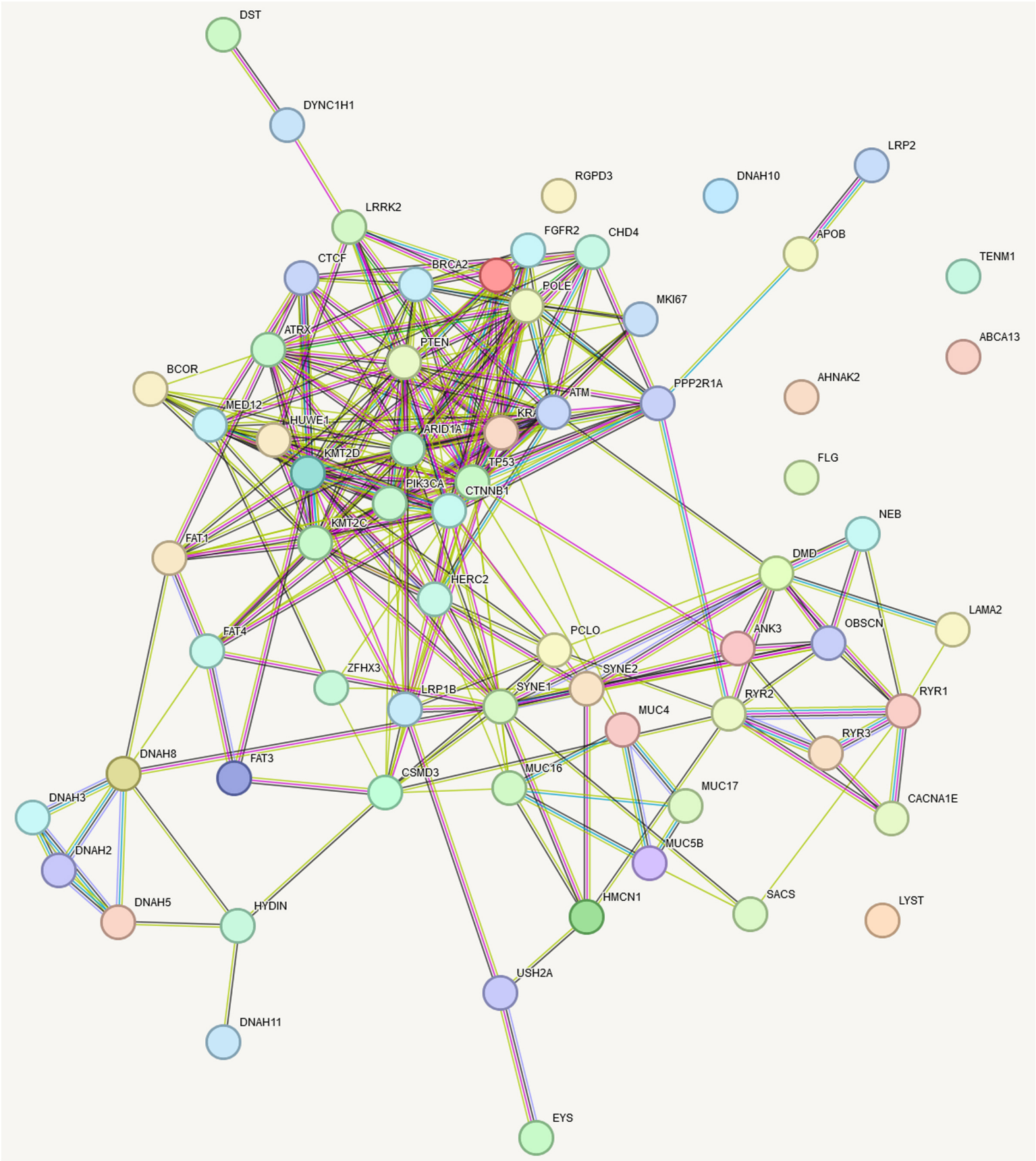

Supplement: S3 Fig — A map of network interactions using STRING database. Please see Materials and methods for this analysis. (PDF) [file pone.0299114.s003.pdf]

Supplemental Figure S5. Mutations observed to effect electrostatic surface potential

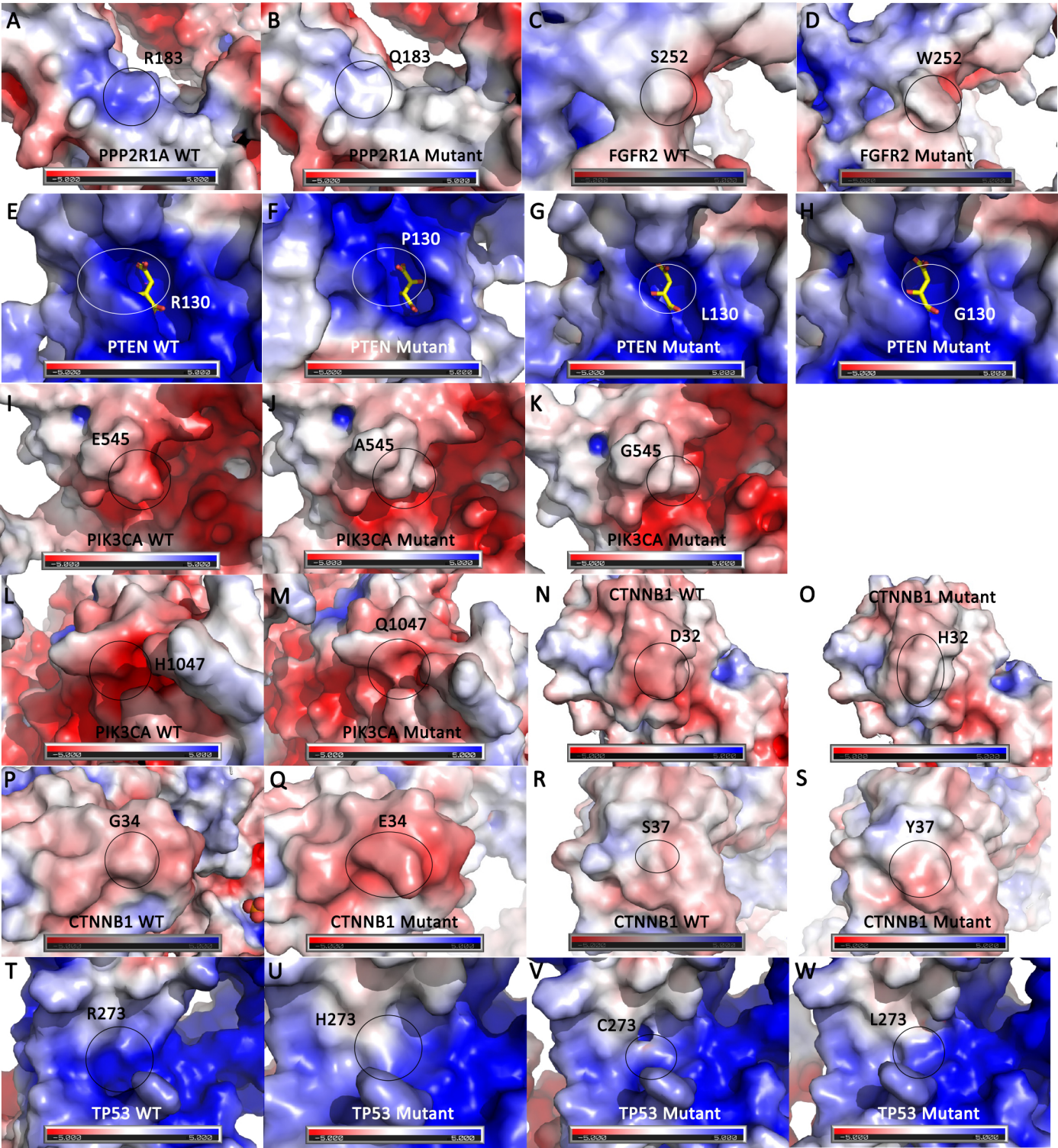

Supplement: S5 Fig — Surface rendering of the protein structure is shown with basic or positive surface potential colored blue, acidic or negative colored red, and neutral colored white. The WT or mutant residue location is identified by a black or white circle. (PDF) [file pone.0299114.s005.pdf]
