# Supplementary material for: Detection of driver mutations and genomic signatures in endometrial cancers using artificial intelligence algorithms
Source: PLoS One. 2024 Feb 26;19(2):e0299114. doi: 10.1371/journal.pone.0299114 (PMC10896512; doi:10.1371/journal.pone.0299114)

Supplemental Figure S4. Comparison of WT and mutant structures where a reduction in tertiary polar interactions was observed

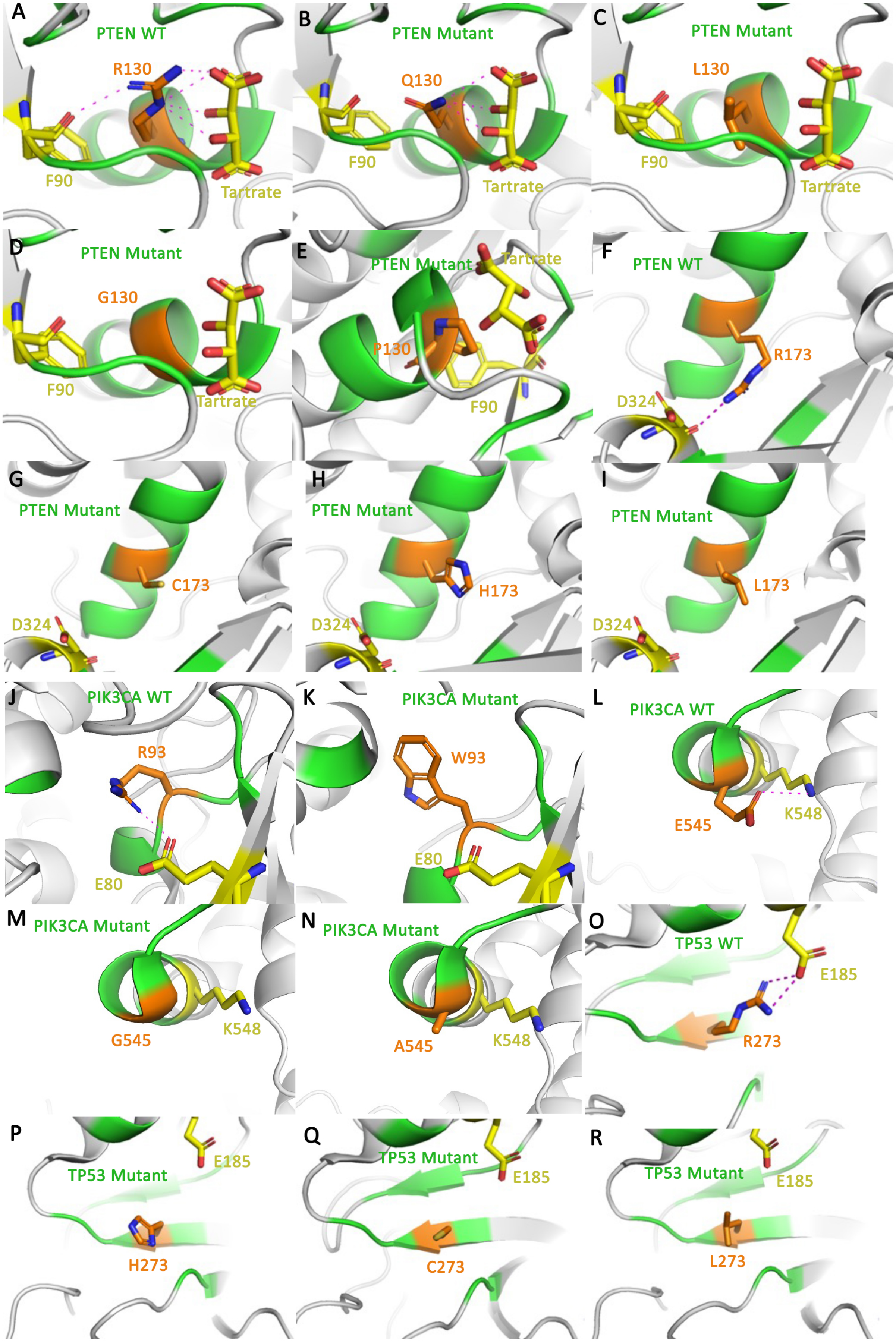

Supplement: S4 Fig — Protein structures are shown with the mutated residue shown in orange. Interacting residues or small molecules are shown in yellow sticks. Polar interactions (salt bridge, hydrogen bond, or dipole-dipole) are shown as magenta dashed lines. (PDF) [file pone.0299114.s004.pdf]
