# Supplementary material for: Detection of driver mutations and genomic signatures in endometrial cancers using artificial intelligence algorithms
Source: PLoS One. 2024 Feb 26;19(2):e0299114. doi: 10.1371/journal.pone.0299114 (PMC10896512; doi:10.1371/journal.pone.0299114)
Supplement: S5 Table — (DOCX) [file pone.0299114.s010.docx]

**Supplementary Table S5.** Previously reported high frequency driver mutations also identified in this study. This is an extended discussion of mutations presented in the main text of the paper. Some references and descriptions may be identical between this table and main text.

| **Gene** | **Description** | **Reference** |
| --- | --- | --- |
| **PPP2R1A** | **Protein phosphatase 2A (PP2A) is a heterotrimeric serine/threonine phosphatase (A, structural subunit; B, regulatory subunit; C, catalytic subunit) which functions primarily in negative regulation of cell division and acts as a tumor suppressor. PPP2R1A encodes one of the structural subunits (the other being PPP2R1B) and has been previously identified as a highly mutated target in ECs as well as other cancers. The P179 residue is the most recurrent mutation identified in PP2R1A. Biochemical and molecular modeling shows that this mutation severely affects its interaction with the C-subunits and disrupts holoenzyme formation and promotes tumor growth. Mutations at R183 and S256 residues are less disruptive.** | **(Shih Ie *et al.* 2011; Jeong *et al.* 2016; Kauko and Westermarck 2018; Remmerie and Janssens 2019; Taylor *et al.* 2019)** |
| **FGFR2** | **FGFR2 is a receptor tyrosine kinase with pleiotropic functions including cell proliferation and mutations have oncogenic potential in many cancers including ECs. Recurrent EC mutations target primarily the S252 and N550 residues. The S252W variant has been demonstrated to affect ligand affinity and specificity. N550K is a dominant activating mutation which affects cell polarity and migration in several cancers including ECs. The receptor has been targeted by FGFR2 single molecule inhibitor infigratinib. Of all the previously reported mutations in ECs we only identified a handful that have the potential to be driver (Fig. 3). The others are predicted to be passenger.** | **(Pollock *et al.* 2007; Gatius *et al.* 2011; Stehbens *et al.* 2018; Huynh *et al.* 2019; Hamid and Petreaca 2020; Willie *et al.* 2022)** |
| **PTEN** | **PTEN is a tumor suppressor phosphatase involved in cell proliferation, migration, survival, apoptosis, and other cell division functions often mutated in many cancers.** Its major function is in regulation of the PI3K/Akt/mTOR pathway where it antagonizes the PI3K signal. Over 80% of ECs have alterations in the PI3K/Akt/mTOR with at least 70% PTEN mutations. Most PTEN cancer mutations including in ECs concentrate in its phosphatase domain and either completely inactivate or downregulate its activity. In addition to the several known hotspot point mutations (R130G, C136F, R173C), **the R130G, R130L, and R130G mutations have been studied in detail and shown to destabilize protein structure and have been implicated in gliomas. The R173C/H mutations have also been shown to negatively impact protein structure and associated with glioma. PTEN is also inactivated by truncations resulting from premature stop codons (Fig.3). Of these, the R130 and R233 residues represent two truncating hotspots of PTEN. R130 is found within the phosphatase domain and inactivates the gene function while R233 truncates the C-2 domain and has been identified in other cancers.** | **(Wang *et al.* 1997; Lee *et al.* 1999; Wang and Jiang 2008; Chi *et al.* 2012; Molinari and Frattini 2013; Markowska *et al.* 2014; Neychev *et al.* 2016; Smith and Briggs 2016; Bell and Ellenson 2019; Li *et al.* 2022; Wu *et al.* 2022; Halling *et al.* 2023)** |
| **PIK3CA** | **PIK3CA is the catalytic subunit of PI3K mentioned above, which functions in the same pathway with PTEN. Both PTEN and PIK3CA occur in ECs and often simultaneously. Several PIK3CA hotspot mutations are activating producing an oncogenic phenotype (E542K, E545K, H1047R) and they occur with high frequency in many cancers, primarily colon, brain and gastric.** | **(Samuels *et al.* 2004; Kang *et al.* 2005; Oda *et al.* 2005; Saal *et al.* 2005)** |
| **CTNNB1** | **CTNNB1 encodes the beta-catenin protein involved in the Wnt-signaling pathway with pleiotropic functions including cell proliferation and migration. Driver mutations hotspots were identified in the N-terminal region of the protein in the COSMIC data. The N-terminus of CTNNB1 is phosphorylated by GSK-3beta at S33, S37, S45, and T41 which leads to beta-catenin degradation. Mutations in this region which cause an oncogenic phenotype, have been previously identified and generally occur in early state ECs.** | **(Gottardi and Gumbiner 2001; Pai *et al.* 2017) (Liu *et al.* 2002; Liu *et al.* 2014; Koelman *et al.* 2022; Ledinek *et al.* 2022; Parrish *et al.* 2022; Travaglino *et al.* 2022)** |
| **TP53** | **TP53 mutations in ECs occur primarily in carcinomas (particularly serous) where they are drivers of cellular transformation. Over 96% of the cancers analyzed here represent endometrioid carcinomas; thus, these data are a representation of TP53 mutations in non-serous histology. Nevertheless, even in endometrial carcinomas, we identified several mutations primarily in the P53 DNA binding domain and two hotspots at R248 an R273. The R248Q mutation was previously described as having an oncogenic phenotype in ECs; the p53-R248Q allele has a higher affinity for the REGY promoter that enhances the activity of the 20S proteosome towards degradation of tumor suppressors. Similarly, the R273 mutation also displays oncogenic phenotypes with various variants showing different oncogenic potential.** | **(Cancer Genome Atlas Research *et al.* 2013; Wang *et al.* 2015; Piulats *et al.* 2017; Bell and Ellenson 2019; Garg *et al.* 2020)** |
| **BCOR** | **BCOR interacts with BCL-6 and acts as a transcriptional co-repressor. It functions primarily in cell differentiation, through modulation of the function of polycomb repressive complex 1 (PRC1). Other functions include pluripotency and cell fate determination. BCOR mutations have been identified in many cancers including endometrial, primarily endometrial stromal sarcomas. In Ewing-like sarcomas as well as other tumors BCOR is also characterized by re-arrangements and fusions with other genes. However, BCOR mutations have been reported in certain endometrial carcinomas and we also identified a previously reported BCOR hotspot (N1459S) in the COSMIC cohort. A preliminary non-peer reviewed report showed that this mutation occurs in 3.2% of TCGA cases.** The N1459 residue is located in a flexible linker region before the ankyrin domain, and this part of the protein has been shown to be involved in interacting with KDM2B. This residue is not located in any of the structured BCOR domains that have been experimentally determined and due to the flexibility of this region, the AlphaFold model has very low confidence in this region of the protein. Therefore, this mutation was not modeled computationally due to the lack of reliable model for this section of the protein. | **(Huynh *et al.* 2000; Cancer Genome Atlas Research *et al.* 2013; Chen 2016; Zhao *et al.* 2016; Garcia-Sanz *et al.* 2017; Astolfi *et al.* 2019; Wong *et al.* 2020)** |
| **KRAS** | **KRAS mutations in ECs are characterized primarily by the canonical activating oncogenic glycine-12 substitution primarily to aspartic acid, alanine and valine. Crystal structure of KRAS G12 mutations have been solved and show that it affects interactions with RAS-GEF.** | **(Ring *et al.* 2017; Sogabe *et al.* 2017; Lin *et al.* 2020)** |

**References**

Astolfi, A., M. Fiore, F. Melchionda, V. Indio, S. N. Bertuccio *et al.*, 2019 BCOR involvement in cancer. Epigenomics 11**:** 835-855.

Bell, D. W., and L. H. Ellenson, 2019 Molecular Genetics of Endometrial Carcinoma. Annu Rev Pathol 14**:** 339-367.

Cancer Genome Atlas Research, N., C. Kandoth, N. Schultz, A. D. Cherniack, R. Akbani *et al.*, 2013 Integrated genomic characterization of endometrial carcinoma. Nature 497**:** 67-73.

Chen, T., 2016 Accurate mutation annotation and functional prediction enhance the applicability of -omics data in precision medicine. Unpublished.

Chi, A. S., T. T. Batchelor, D. Dias-Santagata, D. Borger, C. D. Stiles *et al.*, 2012 Prospective, high-throughput molecular profiling of human gliomas. J Neurooncol 110**:** 89-98.

Garcia-Sanz, P., J. C. Trivino, A. Mota, M. Perez Lopez, E. Colas *et al.*, 2017 Chromatin remodelling and DNA repair genes are frequently mutated in endometrioid endometrial carcinoma. Int J Cancer 140**:** 1551-1563.

Garg, A., J. P. Hazra, M. K. Sannigrahi, S. Rakshit and S. Sinha, 2020 Variable Mutations at the p53-R273 Oncogenic Hotspot Position Leads to Altered Properties. Biophys J 118**:** 720-728.

Gatius, S., A. Velasco, A. Azueta, M. Santacana, J. Pallares *et al.*, 2011 FGFR2 alterations in endometrial carcinoma. Mod Pathol 24**:** 1500-1510.

Gottardi, C. J., and B. M. Gumbiner, 2001 Adhesion signaling: how beta-catenin interacts with its partners. Curr Biol 11**:** R792-794.

Halling, G. C., A. M. Udager and S. L. Skala, 2023 Endometrial, Ovarian, and Peritoneal Involvement by Endometrioid Carcinoma, Yolk Sac Tumor, and Endometriosis: Molecular Evidence for a Shared Precursor. Int J Gynecol Pathol 42**:** 247-253.

Hamid, A. B., and R. C. Petreaca, 2020 Secondary Resistant Mutations to Small Molecule Inhibitors in Cancer Cells. Cancers (Basel) 12.

Huynh, H., L. Y. Lee, K. Y. Goh, R. Ong, H. X. Hao *et al.*, 2019 Infigratinib Mediates Vascular Normalization, Impairs Metastasis, and Improves Chemotherapy in Hepatocellular Carcinoma. Hepatology 69**:** 943-958.

Huynh, K. D., W. Fischle, E. Verdin and V. J. Bardwell, 2000 BCoR, a novel corepressor involved in BCL-6 repression. Genes Dev 14**:** 1810-1823.

Jeong, A. L., S. Han, S. Lee, J. Su Park, Y. Lu *et al.*, 2016 Patient derived mutation W257G of PPP2R1A enhances cancer cell migration through SRC-JNK-c-Jun pathway. Sci Rep 6**:** 27391.

Kang, S., A. G. Bader and P. K. Vogt, 2005 Phosphatidylinositol 3-kinase mutations identified in human cancer are oncogenic. Proc Natl Acad Sci U S A 102**:** 802-807.

Kauko, O., and J. Westermarck, 2018 Non-genomic mechanisms of protein phosphatase 2A (PP2A) regulation in cancer. Int J Biochem Cell Biol 96**:** 157-164.

Koelman, E. M. R., A. Yeste-Vazquez and T. N. Grossmann, 2022 Targeting the interaction of beta-catenin and TCF/LEF transcription factors to inhibit oncogenic Wnt signaling. Bioorg Med Chem 70**:** 116920.

Ledinek, Z., M. Sobocan and J. Knez, 2022 The Role of CTNNB1 in Endometrial Cancer. Dis Markers 2022**:** 1442441.

Lee, J. O., H. Yang, M. M. Georgescu, A. Di Cristofano, T. Maehama *et al.*, 1999 Crystal structure of the PTEN tumor suppressor: implications for its phosphoinositide phosphatase activity and membrane association. Cell 99**:** 323-334.

Li, Q., Z. Li, T. Luo and H. Shi, 2022 Targeting the PI3K/AKT/mTOR and RAF/MEK/ERK pathways for cancer therapy. Mol Biomed 3**:** 47.

Lin, D. I., N. Shah, J. Y. Tse, J. K. Killian, A. Hemmerich *et al.*, 2020 Molecular profiling of mesonephric and mesonephric-like carcinomas of cervical, endometrial and ovarian origin. Gynecol Oncol Rep 34**:** 100652.

Liu, C., Y. Li, M. Semenov, C. Han, G. H. Baeg *et al.*, 2002 Control of beta-catenin phosphorylation/degradation by a dual-kinase mechanism. Cell 108**:** 837-847.

Liu, Y., L. Patel, G. B. Mills, K. H. Lu, A. K. Sood *et al.*, 2014 Clinical significance of CTNNB1 mutation and Wnt pathway activation in endometrioid endometrial carcinoma. J Natl Cancer Inst 106.

Markowska, A., M. Pawalowska, J. Lubin and J. Markowska, 2014 Signalling pathways in endometrial cancer. Contemp Oncol (Pozn) 18**:** 143-148.

Molinari, F., and M. Frattini, 2013 Functions and Regulation of the PTEN Gene in Colorectal Cancer. Front Oncol 3**:** 326.

Neychev, V., S. M. Sadowski, J. Zhu, M. Allgaeuer, K. Kilian *et al.*, 2016 Neuroendocrine Tumor of the Pancreas as a Manifestation of Cowden Syndrome: A Case Report. J Clin Endocrinol Metab 101**:** 353-358.

Oda, K., D. Stokoe, Y. Taketani and F. McCormick, 2005 High frequency of coexistent mutations of PIK3CA and PTEN genes in endometrial carcinoma. Cancer Res 65**:** 10669-10673.

Pai, S. G., B. A. Carneiro, J. M. Mota, R. Costa, C. A. Leite *et al.*, 2017 Wnt/beta-catenin pathway: modulating anticancer immune response. J Hematol Oncol 10**:** 101.

Parrish, M. L., R. R. Broaddus and A. B. Gladden, 2022 Mechanisms of mutant beta-catenin in endometrial cancer progression. Front Oncol 12**:** 1009345.

Piulats, J. M., E. Guerra, M. Gil-Martin, B. Roman-Canal, S. Gatius *et al.*, 2017 Molecular approaches for classifying endometrial carcinoma. Gynecol Oncol 145**:** 200-207.

Pollock, P. M., M. G. Gartside, L. C. Dejeza, M. A. Powell, M. A. Mallon *et al.*, 2007 Frequent activating FGFR2 mutations in endometrial carcinomas parallel germline mutations associated with craniosynostosis and skeletal dysplasia syndromes. Oncogene 26**:** 7158-7162.

Remmerie, M., and V. Janssens, 2019 PP2A: A Promising Biomarker and Therapeutic Target in Endometrial Cancer. Front Oncol 9**:** 462.

Ring, K. L., M. S. Yates, R. Schmandt, M. Onstad, Q. Zhang *et al.*, 2017 Endometrial Cancers With Activating KRas Mutations Have Activated Estrogen Signaling and Paradoxical Response to MEK Inhibition. Int J Gynecol Cancer 27**:** 854-862.

Saal, L. H., K. Holm, M. Maurer, L. Memeo, T. Su *et al.*, 2005 PIK3CA mutations correlate with hormone receptors, node metastasis, and ERBB2, and are mutually exclusive with PTEN loss in human breast carcinoma. Cancer Res 65**:** 2554-2559.

Samuels, Y., Z. Wang, A. Bardelli, N. Silliman, J. Ptak *et al.*, 2004 High frequency of mutations of the PIK3CA gene in human cancers. Science 304**:** 554.

Shih Ie, M., P. K. Panuganti, K. T. Kuo, T. L. Mao, E. Kuhn *et al.*, 2011 Somatic mutations of PPP2R1A in ovarian and uterine carcinomas. Am J Pathol 178**:** 1442-1447.

Smith, I. N., and J. M. Briggs, 2016 Structural mutation analysis of PTEN and its genotype-phenotype correlations in endometriosis and cancer. Proteins 84**:** 1625-1643.

Sogabe, S., Y. Kamada, M. Miwa, A. Niida, T. Sameshima *et al.*, 2017 Crystal Structure of a Human K-Ras G12D Mutant in Complex with GDP and the Cyclic Inhibitory Peptide KRpep-2d. ACS Med Chem Lett 8**:** 732-736.

Stehbens, S. J., R. J. Ju, M. N. Adams, S. R. Perry, N. K. Haass *et al.*, 2018 FGFR2-activating mutations disrupt cell polarity to potentiate migration and invasion in endometrial cancer cell models. J Cell Sci 131.

Taylor, S. E., C. M. O'Connor, Z. Wang, G. Shen, H. Song *et al.*, 2019 The Highly Recurrent PP2A Aalpha-Subunit Mutation P179R Alters Protein Structure and Impairs PP2A Enzyme Function to Promote Endometrial Tumorigenesis. Cancer Res 79**:** 4242-4257.

Travaglino, A., A. Raffone, D. Raimondo, S. Reppuccia, A. Ruggiero *et al.*, 2022 Prognostic significance of CTNNB1 mutation in early stage endometrial carcinoma: a systematic review and meta-analysis. Arch Gynecol Obstet 306**:** 423-431.

Wang, H., W. Bao, F. Jiang, Q. Che, Z. Chen *et al.*, 2015 Mutant p53 (p53-R248Q) functions as an oncogene in promoting endometrial cancer by up-regulating REGgamma. Cancer Lett 360**:** 269-279.

Wang, S. I., J. Puc, J. Li, J. N. Bruce, P. Cairns *et al.*, 1997 Somatic mutations of PTEN in glioblastoma multiforme. Cancer Res 57**:** 4183-4186.

Wang, X., and X. Jiang, 2008 PTEN: a default gate-keeping tumor suppressor with a versatile tail. Cell Res 18**:** 807-816.

Willie, D., G. Holmes, E. W. Jabs and M. Wu, 2022 Cleft Palate in Apert Syndrome. J Dev Biol 10.

Wong, S. J., O. Senkovich, J. A. Artigas, M. D. Gearhart, U. Ilangovan *et al.*, 2020 Structure and Role of BCOR PUFD in Noncanonical PRC1 Assembly and Disease. Biochemistry 59**:** 2718-2728.

Wu, Y., J. Wang, L. Ge and Q. Hu, 2022 Significance of a PTEN Mutational Status-Associated Gene Signature in the Progression and Prognosis of Endometrial Carcinoma. Oxid Med Cell Longev 2022**:** 5130648.

Zhao, S., S. Bellone, S. Lopez, D. Thakral, C. Schwab *et al.*, 2016 Mutational landscape of uterine and ovarian carcinosarcomas implicates histone genes in epithelial-mesenchymal transition. Proc Natl Acad Sci U S A 113**:** 12238-12243.
